# Changes in the prevalence of mental health problems during the first year of the pandemic: a systematic review and dose-response meta-analysis

**DOI:** 10.1136/bmjment-2024-301018

**Published:** 2024-06-13

**Authors:** Georgia Salanti, Natalie Luise Peter, Thomy Tonia, Alexander Holloway, Leila Darwish, Ronald C Kessler, Ian White, Simone N. Vigod, Matthias Egger, Andreas D Haas, Seena Fazel, Helen Herrman, Christian Kieling, Vikram Patel, Tianjing Li, Pim Cuijpers, Andrea Cipriani, Toshi A Furukawa, Stefan Leucht

**Affiliations:** 1 Institute of Social and Preventive Medicine, University of Bern, Bern, Switzerland; 2 Department of Psychiatry and Psychotherapy, School of Medicine and Health, Technical University of Munich, Munich, Germany; 3 Department of Health Care Policy, Harvard Medical School, Boston, Massachusetts, USA; 4 MRC Clinical Trials Unit at UCL, University College London, London, UK; 5 Women’s College Hospital and Department of Psychiatry, Faculty of Medicine, University of Toronto, Toronto, Ontario, Canada; 6 Centre for Infectious Disease Epidemiology and Research, Faculty of Health Sciences, University of Cape Town, Rondebosch, South Africa; 7 Department of Psychiatry, University of Oxford, Oxford, UK; 8 Orygen and Centre for Youth Mental Health, The University of Melbourne, Melbourne, Victoria, Australia; 9 Department of Psychiatry, Universidade Federal do Rio Grande do Sul, Porto Alegre, RS, Brazil; 10 Child & Adolescent Psychiatry Division, Hospital de Clinicas de Porto Alegre, Porto Alegre, Brazil; 11 Department of Global Health and Social Medicine, Harvard Medical School, Boston, Massachusetts, USA; 12 Department of Ophthalmology, University of Colorado Anschutz Medical Campus, Aurora, Colorado, USA; 13 Department of Clinical, Neuro and Developmental Psychology, Amsterdam Public Health research institute, Vrije Universiteit Amsterdam, Amsterdam, Netherlands; 14 International Institute for Psychotherapy, Babeș-Bolyai University, Cluj-Napoca, Romania; 15 Oxford Precision Psychiatry Lab, NIHR Oxford Health Biomedical Research Centre, Oxford, UK; 16 Warneford Hospital, Oxford Health NHS Foundation Trust, Oxford, UK; 17 Office of Institutional Advancement and Communications, Kyoto University, Kyoto, Japan

**Keywords:** COVID-19, Data Interpretation, Statistical, Depression

## Abstract

**Aim:**

To describe the pattern of the prevalence of mental health problems during the first year of the COVID-19 pandemic and examine the impact of containment measures on these trends.

**Methods:**

We identified articles published until 30 August 2021 that reported the prevalence of mental health problems in the general population at two or more time points. A crowd of 114 reviewers extracted data on prevalence, study and participant characteristics. We collected information on the number of days since the first SARS-CoV-2 infection in the study country, the stringency of containment measures and the number of cases and deaths. We synthesised changes in prevalence during the pandemic using a random-effects model. We used dose-response meta-analysis to evaluate the trajectory of the changes in mental health problems.

**Results:**

We included 41 studies for 7 mental health conditions. The average odds of symptoms increased during the pandemic (mean OR ranging from 1.23 to 2.08). Heterogeneity was very large and could not be explained by differences in participants or study characteristics. Average odds of psychological distress, depression and anxiety increased during the first 2 months of the pandemic, with increased stringency of the measures, reported infections and deaths. The confidence in the evidence was low to very low.

**Conclusions:**

We observed an initial increase in the average risk of psychological distress, depression-related and anxiety-related problems during the first 2 months of the pandemic. However, large heterogeneity suggests that different populations had different responses to the challenges imposed by the pandemic.

**WHAT IS ALREADY KNOWN ON THIS TOPIC**

- Previous systematic reviews have drawn varied conclusions regarding the impact of the first year of the pandemic on the prevalence of mental health problems in the general population. These conclusions range from reporting ‘a significant increase’ to noting ‘a small and heterogeneous impact’.

**WHAT THIS STUDY ADDS**

- The significant variability in study findings suggests that certain subgroups of the general population experienced a substantial increase in the prevalence of depression, anxiety and psychological distress symptoms. This increase was most pronounced during the first 2 months of the pandemic, with stricter measures and a rising number of reported infections.

**HOW THIS STUDY MIGHT AFFECT RESEARCH, PRACTICE OR POLICY**

- Our findings should help decision-makers interpret the benefits of governmental containment measures to control the pandemic, considering their generally small impact on the population’s mental health and the short duration of symptom exacerbation.

## Introduction

Mental health is a major public health concern.^1^ Mental health conditions have long been and still are underdiagnosed and undertreated, in particular in low-income and middle-income countries.^2 3^ The COVID-19 pandemic accentuated the need for increased priority to be given to mental health, and much discussion took place about the potential surge of mental health problems as the result of the exacerbation of risk factors such as isolation, uncertainty about the future, disruption of work and education, economic adversities, the fear of sickness and loss of loved ones.^4^

Estimating whether and to what magnitude the prevalence of mental health problems changed with the COVID-19 pandemic has been important, for both gauging the benefits and harms of containment measures and for designing optimal public health interventions to prevent harms in the future. Systematic reviews published on the topic up to now have focused primarily on estimating the changes in prevalence or symptoms of mental health problems during the pandemic compared with prepandemic levels.^5–12^ The body of evidence showed that the impact of the pandemic on the general population’s mental health has been possibly mild to at worst moderate; it also appears to be very heterogeneous. Several individual studies and one systematic review suggest that changes in mental health in the general population of countries are time-dependent, with higher levels of increase in anxiety and depression symptoms in the first 2 months of the pandemic, but not thereafter.^13 14^

The present article is part of the living systematic review project MHCOVID (https://mhcovid.ispm.unibe.ch) that involved and trained a global crowd of researchers to conduct parts of the review process.^15 16^ We conducted a living systematic review of longitudinal studies in the general population published until 31 March 2021. We used dose-response meta-analysis to evaluate the trajectory of the changes in mental health symptom scores during the first months of the pandemic.^13^ Here, we updated our database with studies published until the end of August 2021 and we aimed to investigate how the proportion of people with mental health problems changed over time as a function of the stringency of the governmental containment measures and the numbers of reported deaths and SARS-COV2 cases.

## Methods

### Search strategy and study selection criteria

The protocol of this systematic review was registered with PROSPERO (CRD42020180049).

We searched MEDLINE and EMBASE using the ISPM COVID-19 living evidence database https://ispmbern.github.io/COVID-19/living-review/collectingdata.html. Key words and terms are: for MEDLINE (‘Wuhan coronavirus’ (online supplemental (Supplementary Concept) OR ‘COVID-19’ OR ‘2019 ncov’(tiab) OR ((‘novel coronavirus’(tiab) OR ‘new coronavirus’(tiab)) AND (wuhan(tiab) OR 2019(tiab))) OR 2019-nCoV(All Fields) OR (wuhan(tiab) AND coronavirus(tiab)))))) and for EMBASE ncov OR (wuhan AND corona) OR COVID. We filtered these data on (mental) OR (alcoho*) OR (violen*) OR (subst*) OR (abuse) in title or abstract. A full filtering code is presented online in https://esm-ispm-unibe-ch.github.io/covid19-mhsr/search-strategy/. We included studies fully published until the end of August 2021.

We included population-based studies that reported data on any mental health condition (including alcohol and substance abuse and violence, or a positive mental health outcome such as life satisfaction or mental well-being). These studies included data for at least two distinct time points, with at least one of these time points during the pandemic. Two designs are relevant to these requirements: longitudinal studies of the same individuals assessed at multiple time points and cross-sectional studies in separate samples drawn from the same or comparable populations at multiple time points. Studies were included if they measured the number of people with symptoms using the same instrument (a diagnostic interview or a validated diagnostic or screening questionnaire) with the same cut-off value across the eligible time points.

Studies were required to include participants from the general population irrespective of sex and age (ie, children, adolescents, adults and elderly). We excluded studies undertaken exclusively with participants not representative of the general population: people with a particular condition or health status (eg, diabetics), a particular occupation (healthcare personnel, teachers) or in a special living situation (eg, refugees) and COVID-19 patients. We also excluded studies based on hospital visits and medical records as well as studies that recruited participants via social media as their users are not representative of the general population.

Pairs of investigators from the MHCOVID crowd of 114 health or research professionals independently judged the eligibility of the studies according to detailed written instructions. Disagreements were resolved by arbitration by a third independent reviewer. For the next phase, pairs of 66 investigators received detailed live and asynchronous training and reviewed the main reports and supplementary materials of the included articles using pretested forms in REDCap (Research Electronic Data Capture).^17^ They extracted all relevant information from the included studies and assessed the risk of bias. Any discrepancies were resolved by consensus and arbitration by a panel of investigators within the review team (NLP, TT, GS and LD). In case of missing data or unclear information reported in the articles, we contacted the authors for clarifications.

In the online supplemental appendix, we present the inclusion criteria and the studies selection process in more detail.

10.1136/bmjment-2024-301018.supp1 Supplementary data

### Outcomes, exposures and other data collected.

For each time point and mental health condition, we extracted the count of individuals surpassing a diagnostic or screening threshold using the same diagnostic or screening instrument across time points, out of the total individuals evaluated. We recorded the threshold as reported by the study authors; in case of multiple thresholds reported, we extracted data according to a lowest threshold value. Scales measuring more than one condition (eg, anxiety and depression) or aiming to screen for general symptoms of mental ill health were considered under the term ‘psychological distress’. Since some people benefit from the positive aspects of the situation, such as reduced commuting and more time with family, we also included scales that measure positive mental health features.^15^ We excluded scales developed or modified specifically to assess symptoms caused by the COVID-19 pandemic, as we did not consider the results comparable to prepandemic measurements. Risk of bias in the included studies was measured using a tool we developed for the purpose of this meta-analysis.^18^ The tool evaluates the risk of selection bias (sample invited and/or sample providing data are not representative of the general population) and information bias (associated with the measurement of the condition).

We considered four variables and examined their impact on the changes in mental health. For every time point reported we considered publicly available data from the study country to define the following variables.

Time in the pandemic: Number of days elapsed between the official recording of the first SARS-Cov-2 in the study country and the time point of study data.

Stringency of the containment measures: An index (–100) representing the stringency of government containment and closure policies, economic policies and health system policies as provided by the Oxford COVID-19 Government Response Tracker.^19^

Cumulative cases: The cumulative number of the SARS-Cov-2 confirmed reported cases since the registration of the first case.

Cumulative deaths: The cumulative number of reported COVID-19-related deaths since the registration of the first case.

We also collected data on variables that might be associated with changes in mental health symptoms and people’s resilience; study characteristics (such as method of recruitment, country of study, study design) and participant characteristics (mean age, percentage of females, etc) and study country characteristics (gross domestic product (GDP) per capita and the Gini Inequality Index in 2019).

### Statistical analysis

Within each study, we used the number of people with symptoms above the reported threshold to calculate ORs between the earliest reported time point and any subsequent time points. The ORs in studies reporting more than two time points are correlated. We accounted for these correlations by decreasing the SE of the ORs as previously described.^20^

For the ‘pre vs during’ meta-analysis, we synthesised the data that referred to changes from prepandemic time points using random-effects models. We present the summary ORs for each condition, with its 95% CI and 95% prediction interval.^21^ The amount of heterogeneity was estimated by considering the heterogeneity standard deviation ( τ ) and the width of the prediction interval. We extended the model into a meta-regression to explore the role of other factors that potentially influence the change in outcome, such as mean age, percentage of women, the scale used to measure symptoms severity, country GDP and Gini index and risk of bias. We explored sensitivity to the employed effect metric (using risk ratio instead of OR). The importance of each covariate in meta-regression was evaluated by considering the 95% CI of the regression coefficient (how compatible is with zero) and the change in the estimated heterogeneity compared with the meta-analysis model. For conditions with at least 10 comparisons, we draw contour-enhanced funnel plots to explore the potential presence of small study effects and reporting bias.

The ‘dose-response’ meta-analysis related the OR for the studied conditions to each of the four variables described in the previous section. We employed a one-stage dose-response meta-analysis model with random effects and restricted cubic splines for the exposure variable, with three knots placed by default at the 20th, 50th and 80th quintiles.^22 23^ The cumulative cases and deaths were log-transformed before entering the model. We performed sensitivity analyses to the location of the knots.

All models were fitted in R using the libraries meta and dosresmeta.^24 25^

### Evaluating the confidence in the evidence

For the pre-during meta-analysis, we used the Grading of Recommendations Assessment, Development and Evaluation (GRADE) approach to evaluate the confidence in the evidence synthesis results adapting guidance for prognostic studies.^26 27^

### Data sharing

The full data set is freely available online in BORIS (Bern Open Repository and Information System, www.boris.unibe.ch) and are assigned a permanent and unique digital object identifier (DOI, https://doi.org/10.48620/403). The analysis code and data are available in the GitHub directory https://github.com/esm-ispm-unibe-ch-REPRODUCIBLE/MHCOVID2024-Changes-in-the-prevalence-of-mental-health-problems. The directory also includes two R notebook files (.Rmd) that can be used to reproduce the results section and the online supplemental appendix, also published in http://rpubs.com/geointheworld/Results_MHCOVID_dichotomous and https://rpubs.com/geointheworld/APPENDIX_MHCOVID_dichotomous

## Results

**Figure 1:**
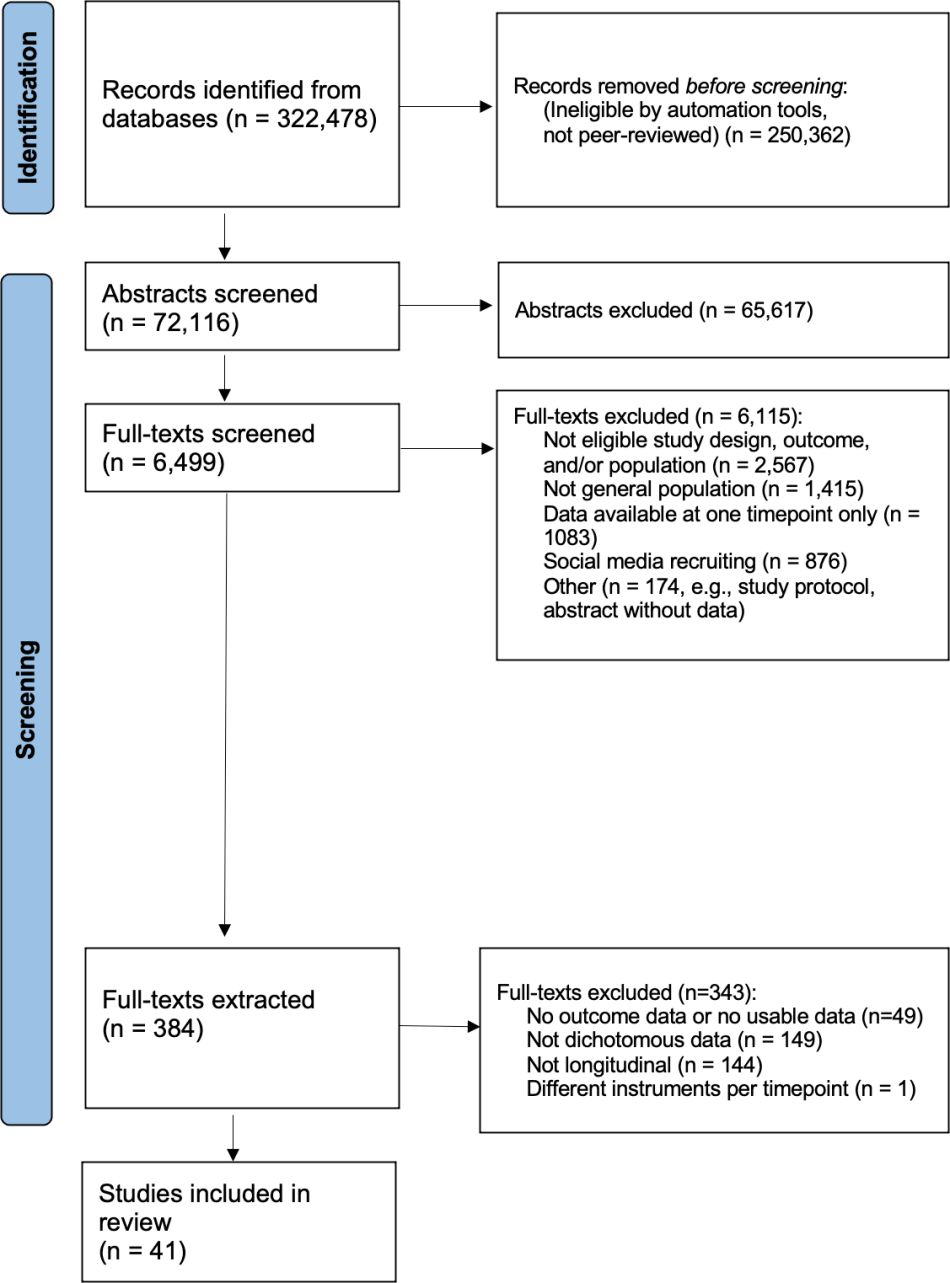
Preferred Reporting Items for Systematic review and Meta-Analysis flowchart of eligible studies.

The flow of study selection is shown in figure 1. Overall, we included 41 studies with data from 750 728 observations about 7 conditions and 123 time points (see online supplemental appendix for a full list of the included studies and their characteristics). Only 12 of those studies were part of our previous systematic review. The studies were conducted in 14 different countries. The prepandemic data were collected as early as 2014, while the most recent data were from January 2021. The median sample size across the 123 time points was 2008 participants. The median of the mean participant age was 44 years (ranged from 5 to 72 years), and over half of the participants across all studies were women. The cumulative COVID-19 cases and deaths, the stringency, economic support and containment and health indices varied widely across time points (table 1). Most studies were repeated cross-sectional surveys. The risk of an unrepresentative sample and non-response bias was high or unclear in most studies. In contrast, most studies showed a low risk of information bias (table 2).

**Table 1:** Characteristics of study participants, countries and the COVID-19 pandemic. Cumulative cases and deaths are per 100 000 people, averaged over 123 time points reported in 41 studies

	Median	Min	Max	
No of participants	2008	38	90 798	
Mean age (years)	44	5	72	
Time points examined	2	1	6	
Percentage of women (%)	54	50	80	
GDP per capita in US$	46 406	11 371	70 920	
Gini index	36	25	52	
Days since first case	99	1	340	
Stringency	73	29	94	
Cumulative cases	172	0	2701	
Cumulative deaths	19	0	75	

GDP, gross domestic product.

**Table 2:** Characteristics of studies

	Number of studies (k=41)	Number of time points (k=123)
Population		
Children	1	2
Children, adolescents	1	4
Adolescents	31	91
Adolescents, adults	2	6
Adults	2	8
Elderly	4	12
Condition		
ADHD	1	2
Alcohol/substance abuse	4	13
Anxiety	13	28
Depression	14	30
Mental well-being	3	6
Psychological distress	13	30
Sleep disturbance	7	14
Design		
Cross-sectional at multiple time points	26	72
Longitudinal	15	51
Risk of unrepresentative sample		
High risk	13	43
Low risk	15	44
Unclear risk	13	36
Risk of information bias		
High risk	5	12
Low risk	35	107
Unclear risk	1	4
Risk of non-response bias		
High risk	20	60
Low risk	9	22
Unclear risk	12	41
Country		
Chile	1	4
China	6	16
Czechia	1	2
Ecuador	1	2
Germany	2	6
Hong Kong	1	4
Iran	1	2
Italy	1	2
Japan	2	5
Netherlands	2	8
Spain	1	2
Switzerland	1	2
UK	10	33
USA	11	35

ADHD, attention deficit hyperactivity disorder.

### Meta-analysis of prepandemic versus during-pandemic prevalence of mental health problems

**Figure 2:**
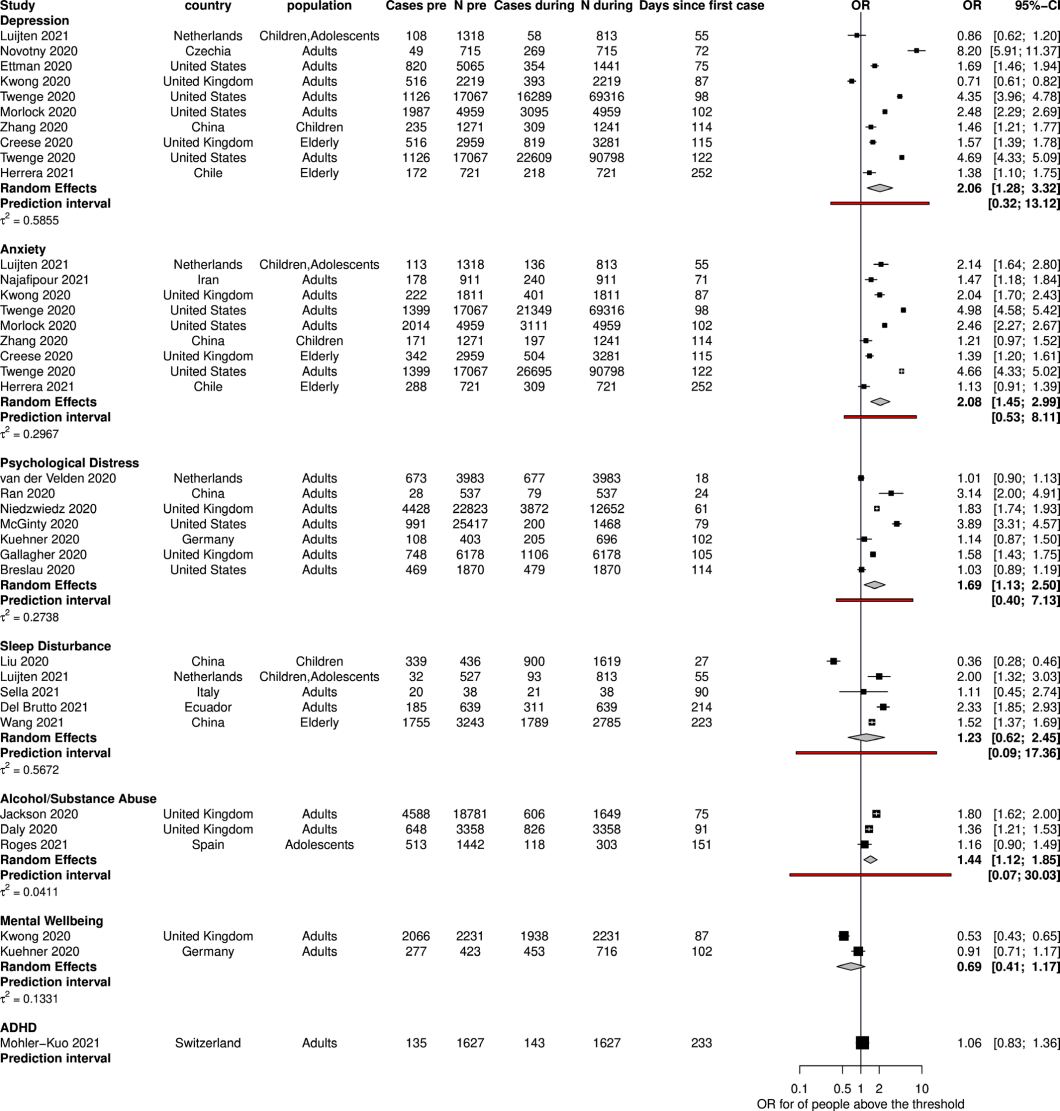
Meta-analysis of ORs for people above a threshold on a symptom scale during the pandemic compared with before the pandemic. OR>1 means that the odds of people above the threshold are larger during the pandemic and hence the average mental health of the population deteriorated.

Of the 41 included studies, 25 provided measurements before and during the pandemic and contributed 37 ORs to the pre-during meta-analysis. The summary ORs for each condition are shown in figure 2.

There was substantial heterogeneity in all conditions, as shown in the width of the prediction intervals. We found no evidence that heterogeneity could be explained by differences in the study methods and country characteristics: the scale used to measure the symptoms, age, sex, GDP per capita, Gini Inequality Index or the study of risk of bias (online supplemental appendix). There was limited evidence that the use of short versions of scales instead of longer (eg, using Patient Health Questionnaire PHQ-2 instead of PHQ-9) was associated with larger ORs (eg, OR 5.29 from three studies using PHQ-2 vs 1.76 from four studies using PHQ-9). There was not a clear indication of small-study effects in depression according to the funnel plot (online supplemental appendix).

Because of large heterogeneity and preponderance of high risk of bias studies, the evidence is judged to be of low confidence for psychological distress, depression and anxiety and very low for sleep disturbances, alcohol/substance abuse and mental well-being (because of additional high uncertainty due to few available studies).

### Dose-response meta-analysis

**Figure 3:**
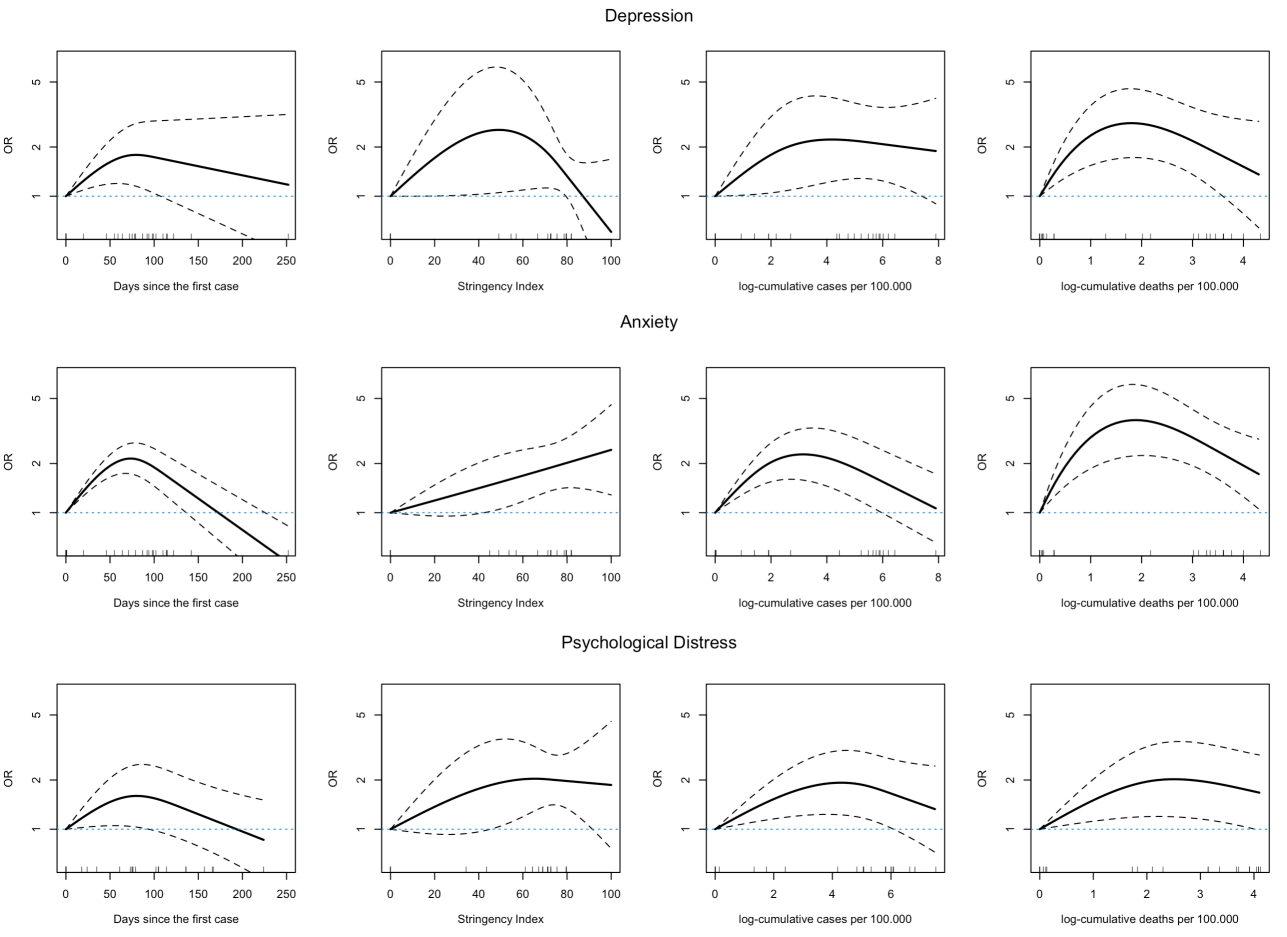
Dose-response meta-analysis plots of the ORs for depression, anxiety and psychological distress as a function of the days since the days of the first case in the study country, the stringency index, the cumulative number of cases and the cumulative number of deaths. CIs are shown as dashed lines. Larger values of OR mean more people above the threshold.

Dose-response meta-analyses were solely performed for anxiety (13 studies), depression (14 studies) and psychological distress (12 studies), as only these conditions provided sufficient data spanning various time points. Figure 3 shows the trajectory of the OR as a function of the time in the pandemic, the stringency index, the cumulative number of cases and the cumulative number of deaths. The odds of having symptoms score above a threshold increased, on average, during the first 2 months after the first reported cases for the three conditions; thereafter they decrease or remain at a stable level but with large uncertainty. The odds of mental health problems also increased with a cumulative number of cases and deaths reported, mostly presenting a non-linear association: the odds increased up to a point and then either decrease, on average or remain stable. There was little evidence that the odds of increased problems with depression and anxiety increased after 60 reported cases and 10 deaths per 100 000 people. The odds of mental health issues also increased with greater stringency, although the shape is not consistent across the conditions.

Sensitivity analyses excluding studies with much longer follow-up than the other studies (post hoc) and after changing the location of the knots in the splines did not materially change the dose-response shapes (see online supplemental appendix). There were not enough studies to allow meaningful subgroup analyses for study-specific and population-specific characteristics.

## Discussion

In this systematic review of 41 studies comprising 750 728 observations from people on 123 time points about 7 different mental health conditions, we found that the impact of the pandemic and of the stringency measures to contain the spread of the virus influenced people’s mental health in a way that varied considerably with time and across populations. This substantial heterogeneity in our data could not be attributed to observed population characteristics (such as age, sex) or country characteristics (social inequalities or the GDP per capita). Consistent with previous findings, the odds of psychological distress and depression and anxiety problems increased within the first 2 months of the pandemic; however, the subsequent trajectory suggested an improvement in the mental health of the population.^13^ Similarly, the general population’s mental health was only initially impacted by news of increased cases and deaths. We also found that stringency measures linearly increase the likelihood of anxiety issues, whereas the odds of depression and psychological distress rise with restrictions, although their severity does not appear to significantly impact these outcomes. As heterogeneity was large in all associations, confidence in these findings ranges from low to very low.

Although the odds of the increase in mental health problems are large (OR between 1.5 and 2.5), this represents, in most settings and populations, a small average change in symptom severity.^13^ Symptom scores above a particular threshold do not equate to a diagnosis of a condition. In most studies, screening tools rather than diagnostic tools were used, and we extracted data according to the lowest threshold reported. Other studies examining longitudinal changes in scores also found small or moderate deterioration in the general population’s mental health.^5 10 13^ Even a small deterioration in depression and anxiety symptoms, however, can impact public health at the population level in a significant way when it involves the entire population, as was the case during the pandemic.

This is our second article that uses dose-response meta-analysis to examine the impact of pandemic and confinement characteristics on the changes in the prevalence of mental health problems, using an updated database and examining a dichotomous outcome. Our previous work synthesised data on continuous changes in symptoms measured by validated scales in 43 longitudinal studies.^13^ Only 12 of those 43 studies reported a dichotomous outcome and were included in this article, yet the main conclusions from our two articles agree to a large extent. The current review encompasses studies published up until August 2021, prior to the widespread rollout of vaccination programmes. As of August 2021, approximately 40% of Europe’s population was vaccinated, compared with just 12% globally, according to https://ourworldindata.org/covid-vaccinations. Interpretation of changes in the prevalence of mental health symptoms post-August 2021 must consider additional factors beyond the stringency of measures and reported cases. These factors include vaccination coverage, social tensions and polarisation concerning the mandatory nature of vaccination in some countries.

In this article, we fitted a non-linear dose-response curve within a study and then pooled the shape characteristics across studies. This technique is more powerful than meta-regression and allows us to incorporate non-linear associations. This is important, as several of the exposure variables are unlikely to have a linear effect on the changes in mental health. In contrast to meta-regression, our model can incorporate data from cohorts recruited and assessed repeatedly during the pandemic, increasing the precision of the estimated trajectory curves. Our review is also the first one, to our knowledge, to crowdsource the screening and data extraction process at such a large, global scale. This group of dedicated volunteers has been instrumental in processing the very large number of studies published so far.

Our study has some limitations. The dates of data collection were not always precisely reported in the studies, and this type of measurement error could affect the association between mental health, stringency measures and number of cases and deaths. Second, we studied changes in the number of participants above the minimum reported threshold on a symptoms scale; consequently, the observed odds are overestimations of the odds of depression, anxiety and psychological distress. Third, studies from low and middle-income countries were under-represented in our review, and we excluded studies with participants with pre-existing mental or physical conditions. Robinson *et al* found that the increase in symptoms was more pronounced in samples with physical conditions,^5^ and Sun *et al* found that anxiety and depression deterioration were more pronounced in women,^10^ although these conclusions are challenged by the large heterogeneity found. Risky, prepandemic behaviour, caregiving responsibilities and poor mental health were found to be associated with increased substance and alcohol use in a narrative review.^6^ There are broader concerns that the mental health of vulnerable population subgroups worsened during the pandemic.^28^ Comparisons with disasters at other times and places suggest the mental health of a general population tends to worsen and then returns to baseline over weeks or months, but that this worsening persists for about 20% of the population: members of socio-economically disadvantaged or marginalised groups, and those who have experienced multiple traumas. We focused on short-term impacts of the pandemic and the associated societal measures on the mental health of the total population. We cannot rule out the possibility that the pandemic will have a long-term adverse effect on mental health globally. There is already evidence suggesting that COVID-19 can affect developing brains and minds.^29^ We need to continue to assess and measure the magnitude of such consequences as well as the effects on people already living with adversity.

Prior systematic reviews fell short in accounting for heterogeneity in their conclusions or investigating temporal changes in mental health. The overall conclusions from the Global Burden of Disease project indicated that ‘the impacts on the prevalence and burden of major depressive disorder and anxiety disorders were substantial, which contrasts with our findings of a moderate decline in mental health, primarily observed in the first 2 months of the pandemic.^9^ We were unable to identify any methodological differences that could explain these diverging conclusions, owing to the absence of publicly accessible data, analysis scripts and detailed methodologies in a study by Santomauro *et al*.^9^ This highlights the importance of adhering to the principles of findability, accessibility, interoperability, and reusability (FAIR principles) in the planning, analysis and publication of systematic reviews.^30^ Our review underscores the significant heterogeneity present in mental health studies. A primary strength lies in our ability to quantify substantial heterogeneity by assessing the between-studies variance of the variables of interest. We have presented this heterogeneity, when synthesis was deemed appropriate, through prediction intervals. Furthermore, we have downgraded the certainty of evidence following the principles of the GRADE framework. Meta-regression, previously used to examine the association between time and mental health changes, inappropriately assumes a linear shape and is less powerful than dose-response meta-analysis.^5 9^ These factors likely account for their failure to detect any evidence of the association between changes in mental health and stringency or the number of reported cases and deaths.^5 9^

Our review reveals that a decline in mental health was observed in the first 2 months of the pandemic, coinciding with the reporting of the first SARS-CoV-2 cases and deaths. Subsequently, our findings suggest that various populations swiftly adapted to the new conditions of life. Following the initial shock and upsurge in mental health problems, signs of recovery to prepandemic levels became evident. These observations should be considered from broader societal, political and economic perspectives and should be weighed against the certainty surrounding the distancing measures that efficiently contained the spread of the virus, decongested the hospitals and saved lives.^31 32^

## Data Availability

Data are available in a public, open access repository. The full data set is freely available online in BORIS (Bern Open Repository and Information System, www.boris.unibe.ch) and are assigned a permanent and unique digital object identifier (https://doi.org/10.48620/403). The analysis code and data are available in the GitHub directory https://github.com/esm-ispm-unibe-ch-REPRODUCIBLE/MHCOVID2024-Changes-in-the-prevalence-of-mental-health-problems. The directory also includes two R notebook files (.Rmd) that can be used to reproduce the results section and the appendix, also published in http://rpubs.com/geointheworld/Results_MHCOVID_dichotomous and https://rpubs.com/geointheworld/APPENDIX_MHCOVID_dichotomous
