# Supplementary material for: Changes in the prevalence of mental health problems during the first year of the pandemic: a systematic review and dose-response meta-analysis
Source: BMJ Ment Health. 2024 Jun 13;27(1):e301018. doi: 10.1136/bmjment-2024-301018 (PMC11177678; doi:10.1136/bmjment-2024-301018)
Supplement: Supplementary data [file bmjment-2024-301018supp001.pdf]

APPENDIX

Changes in prevalence of mental health problems during the first year of the COVID 19 pandemic a systematic review and dose-response meta-analysis with associated control measures

Table of Contents

1 Inclusion and exclusion criteria and study selection 2
1.1 Inclusion criteria 2
1.2 Abstract, full-text screening and data extraction 3
2 Exploration of heterogeneity in pre-during analysis 4
2.1 Using risk ratio as a summary measure 4
2.2 The use of different scales in depression, anxiety and psychological distress 4
2.3 Sensitivity analyses including only adult populations and low risk of bias studies 6
2.4 Funnel Plot 8
2.5 Meta-regression in studies for anxiety, depression, and psychological distress 8
3 Results from dose-response meta-analysis 9
3.1 Dose-response depression as a function of days since the first recorded case 9
3.2 Dose-response depression as a function of stringency index 10
3.3 Dose-response depression as a function of log-cases per 10.000 people 10
3.4 Dose-response depression as a function of log-deaths per 10.000 people 11
3.5 Dose-response anxiety as a function of days since the first recorded case 12
3.6 Dose-response anxiety as a function of stringency index 12
3.7 Dose-response anxiety as a function of log-cases per 10.000 people 13
3.8 Dose-response anxiety as a function of log-deaths per 10.000 people 14
3.9 Dose-response psychological distress as a function of days since the first recorded case 14
3.10 Dose-response psychological distress as a function of stringency index 15
3.11 Dose-response psychological distress as a function of log-cases per 10.000 people 15
3.12 Dose-response psychological distress as a function of log-deaths per 10.000 people 16
4 Sensitivity analysis for dose-response meta-analysis 17
4.1 Excluding a study with very long follow-up 17
4.2 Sensitivity to knot locations 18
4.3 Knots at 30%, 50% and 70% quintiles 18
4.4 Knots at 10%, 50% and 90% quintiles 20
5 Included studies and their characteristics. 23
6 Changes to the protocol 26
7 Examples of excluded studies 26

Salanti G, *et al. BMJ Ment Health* 2024; 27:e301018. doi: 10.1136/bmjment-2024-301018

1 Inclusion and exclusion criteria and study selection

1.1 Inclusion criteria

We included original population-based studies. We excluded reviews, commentaries, letters to the editor or editorials (unless they reported results of original research), case reports and preprints. The included studies had to provide information on the prevalence of a defined mental health condition (including, but not limited to depression, anxiety, post-traumatic stress disorder, alcohol or substance use disorder or positive mental health outcomes. We excluded papers that provided information only on health care personnel, or only on people infected with SARS-CoV-2, ill with COVID-19 or COVID-19 survivors.

Studies assessing exclusively personality traits, patterns of emotional reactions (e. g. caregiver burden, parent-child relationship, positive/negative affect, social connectedness, coping, burnout, resilience), loneliness or stress were excluded, as for our analysis, these were not considered defined mental health conditions.

Eligible conditions
Psychological distress
Depression
Anxiety disorders, including:

- Generalized Anxiety Disorder
- Panic Disorder
- Hypochondria/Health Anxiety
- Social anxiety
- Phobia

Suicidality/suicidal thoughts or behaviours/non-suicidal self-harm
Violent behaviour
Acute stress disorder, posttraumatic stress disorder
Somatoform disorder/somatization
Obsessive-compulsive disorder
Paranoid ideation/psychoticism
Substance use disorder, including alcohol use disorder
Non-substance related addictions, including:

- Internet gaming disorder
- Social media addiction/problematic social media use
- Internet addiction
- Gambling disorder
- Problematic smartphone-application use

Insomnia/Sleep disturbance/Sleep quality
Eating disorder
Conduct disorder
Mental Wellbeing
Life Satisfaction
Quality of Life, health-related quality of life

Non-eligible conditions
Any scale specific to COVID-19

Anger
Coping Style
Dementia/Cognition
Expectations regarding aging
Fear
Hostility
Impulsiveness
Loneliness
Mood
Neurasthenia
(Parent-child) relationship quality
Perceived stress
Physical health
Resilience
Sense of Adequacy
Tobacco Use

1.2 Abstract, full-text screening and data extraction

The screening and data extraction tasks were carried out by members of the MHCOVID Crowd: 114 volunteers from 28 different countries, with experience in mental health research and/or systematic reviews, who were trained to carry out these tasks using the REDCap web-application (https://redcap.ispm.unibe.ch). You can find out more about our Crowd and their training here https://mhcovid.ispm.unibe.ch/crowd.html. The crowd investigators screened the abstracts of all studies identified by the search in duplicate and decided about their inclusion according to the following criteria. Disagreements were resolved by arbitration by a third independent reviewer (LD and NP).

For full-text screening and data extraction, the researchers formed fixed pairs, in which both assessed a number of studies and then reviewed the other partner's work. Any discrepancies were resolved by discussion between the pair of extractors and consensus and arbitration by a panel of investigators within the review team.

Using an algorithm described below, the paper was included if:

- All criteria described in section *1.1* were fulfilled.
- At least one eligible outcome was measured using a validated scale or interview (validated meaning the authors provided a verifiable reference for it). Single-item scales and (not separately validated) modified scales were excluded. We excluded scales developed or modified specifically to assess symptoms caused by the COVID-19 pandemic as they lack pre-pandemic measurements and/or were not validated.
- The paper presented findings for the general population (i.e., the whole population of a country/region/city or broad subgroups based on age and gender (e.g., only women or only children)). We excluded studies undertaken exclusively with participants not representative of the general population: people with a particular condition or health status (e.g., diabetics, pregnant women etc.), a particular occupation (teachers, university students) or in a special living situation (e.g., refugees).

- Data was presented for at least two different timepoints (with at least one timepoint during the pandemic). As a result, two study designs were included: longitudinal studies and cross-sectional studies in samples drawn from the same or comparable populations at multiple time points.
- Social media was not amongst the methods used to recruit participants.
- Articles in language other than English were assigned for screening (and extraction later, if eligible) to a member of the crowd that speaks that language.

2 Exploration of heterogeneity in pre-during analysis

2.1 Using risk ratio as a summary measure

The use of risk ratio (RR) instead of odds ratio (OR) did not materially impact on the conclusions from the pre-during analysis. The impact of the pandemic on mental health appears, on average, smaller when RR is used, bu the heterogeneity is large with either measure.

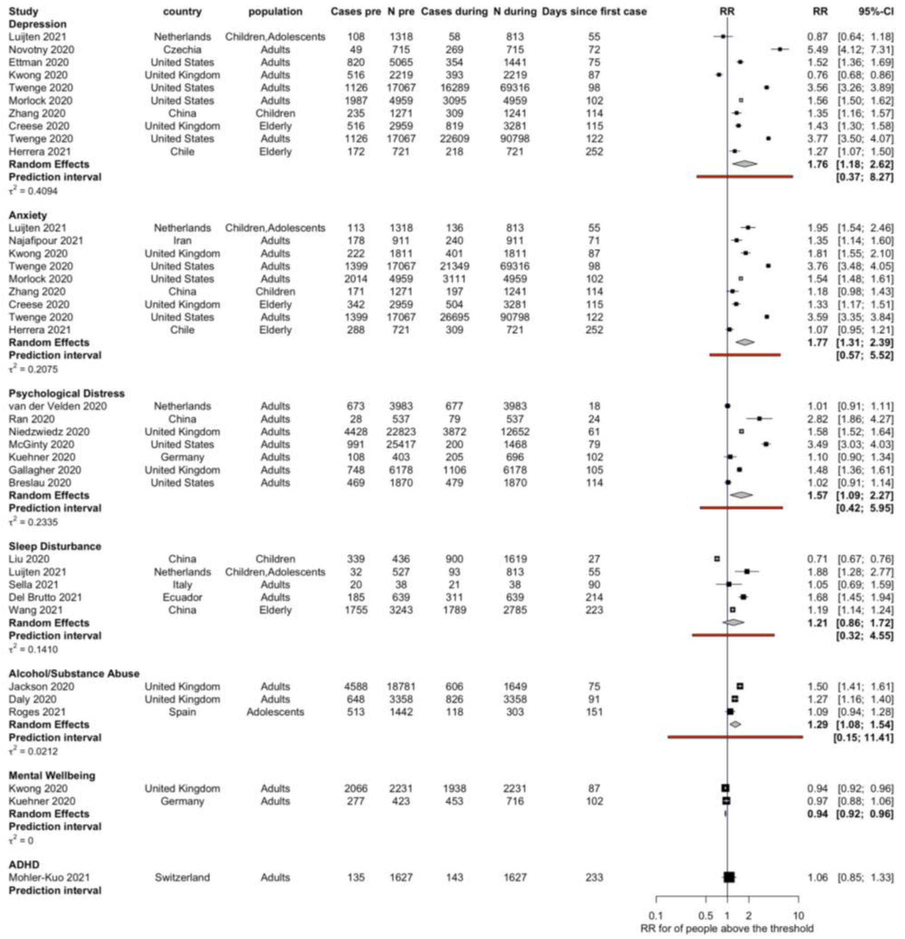

2.2 The use of different scales in depression, anxiety and psychological distress

Several different scales with different thresholds have been used across the studies. This could contribute to heterogeneity. In the following forest plot we present the studies for the three most commonly studied conditions (depression, anxiety and psychological distress) synthesized by the scale they used to measure symptoms severity.

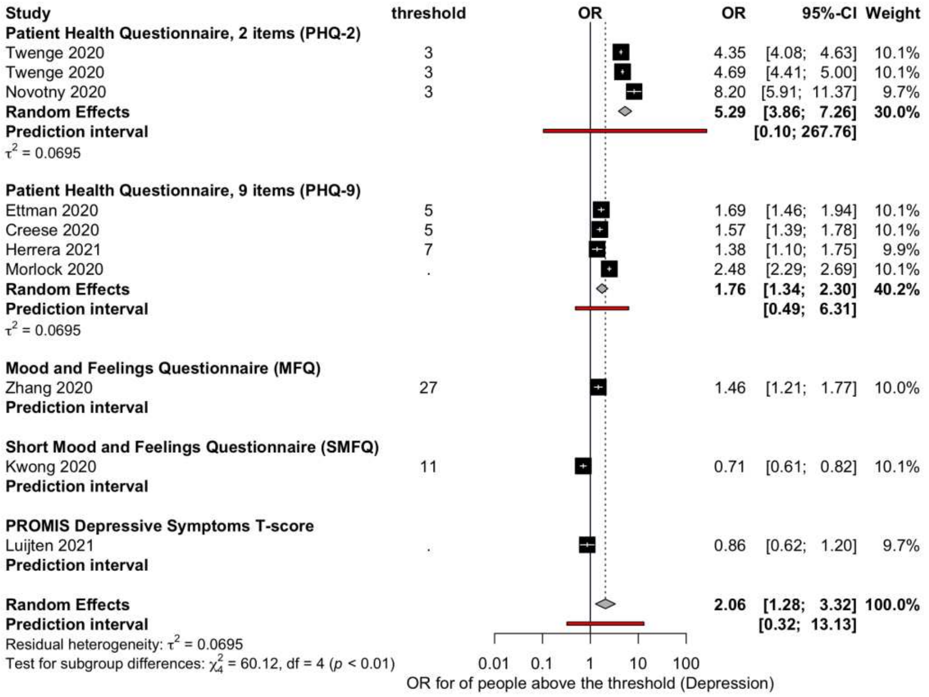
Study
threshold
OR
OR
95%-CI
Weight
Patient Health Questionnaire, 2 items (PHQ-2)
Twenge 2020 3 4.35 [4.08; 4.63] 10.1%
Twenge 2020 3 4.69 [4.41; 5.00] 10.1%
Novotny 2020 3 8.20 [5.91; 11.37] 9.7%
Random Effects 5.29 [3.86; 7.26] 30.0%
Prediction interval [0.10; 267.76]
τ² = 0.0695
Patient Health Questionnaire, 9 items (PHQ-9)
Ettman 2020 5 1.69 [1.46; 1.94] 10.1%
Creese 2020 5 1.57 [1.39; 1.78] 10.1%
Herrera 2021 7 1.38 [1.10; 1.75] 9.9%
Morlock 2020 . 2.48 [2.29; 2.69] 10.1%
Random Effects 1.76 [1.34; 2.30] 40.2%
Prediction interval [0.49; 6.31]
τ² = 0.0695
Mood and Feelings Questionnaire (MFQ)
Zhang 2020 27 1.46 [1.21; 1.77] 10.0%
Prediction interval
Short Mood and Feelings Questionnaire (SMFQ)
Kwong 2020 11 0.71 [0.61; 0.82] 10.1%
Prediction interval
PROMIS Depressive Symptoms T-score
Luijten 2021 . 0.86 [0.62; 1.20] 9.7%
Prediction interval
Random Effects 2.06 [1.28; 3.32] 100.0%
Prediction interval [0.32; 13.13]
Residual heterogeneity: τ² = 0.0695
Test for subgroup differences: χ²₄ = 60.12, df = 4 (p < 0.01)
0.01 0.1 1 10 100
OR for of people above the threshold (Depression)

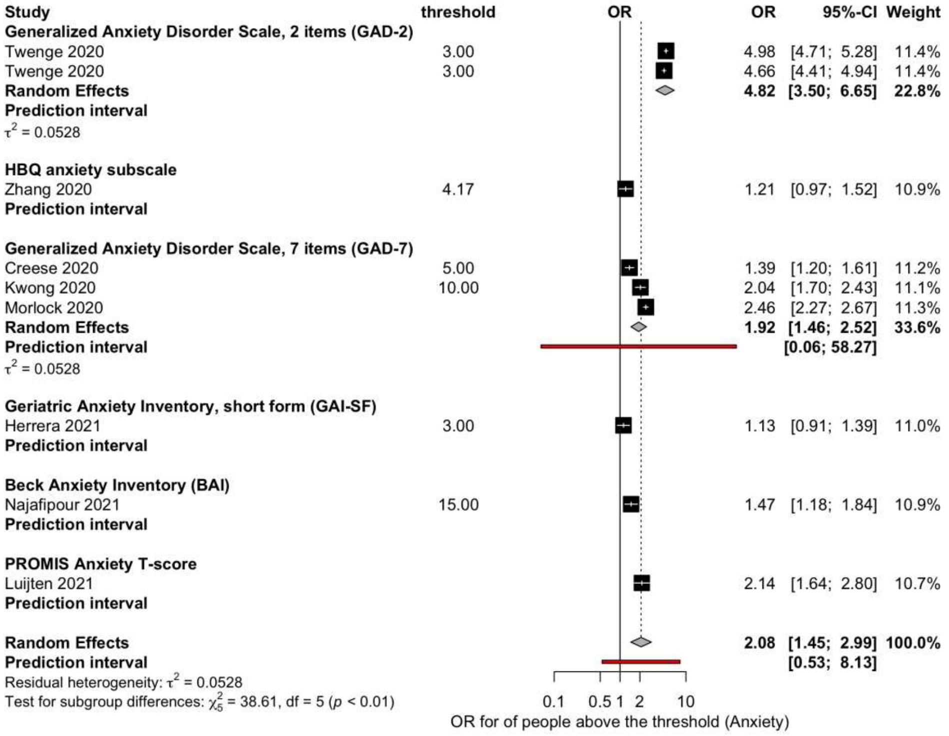
Study
threshold
OR
OR
95%-CI
Weight
Generalized Anxiety Disorder Scale, 2 items (GAD-2)
Twenge 2020 3.00 4.98 [4.71; 5.28] 11.4%
Twenge 2020 3.00 4.66 [4.41; 4.94] 11.4%
Random Effects 4.82 [3.50; 6.65] 22.8%
Prediction interval
τ² = 0.0528
HBQ anxiety subscale
Zhang 2020 4.17 1.21 [0.97; 1.52] 10.9%
Prediction interval
Generalized Anxiety Disorder Scale, 7 items (GAD-7)
Creese 2020 5.00 1.39 [1.20; 1.61] 11.2%
Kwong 2020 10.00 2.04 [1.70; 2.43] 11.1%
Morlock 2020 2.46 [2.27; 2.67] 11.3%
Random Effects 1.92 [1.46; 2.52] 33.6%
Prediction interval [0.06; 58.27]
τ² = 0.0528
Geriatric Anxiety Inventory, short form (GAI-SF)
Herrera 2021 3.00 1.13 [0.91; 1.39] 11.0%
Prediction interval
Beck Anxiety Inventory (BAI)
Najafipour 2021 15.00 1.47 [1.18; 1.84] 10.9%
Prediction interval
PROMIS Anxiety T-score
Luijten 2021 2.14 [1.64; 2.80] 10.7%
Prediction interval
Random Effects 2.08 [1.45; 2.99] 100.0%
Prediction interval [0.53; 8.13]
Residual heterogeneity: τ² = 0.0528
Test for subgroup differences: χ²₅ = 38.61, df = 5 (p < 0.01)
0.1 0.5 1 2 10
OR for of people above the threshold (Anxiety)

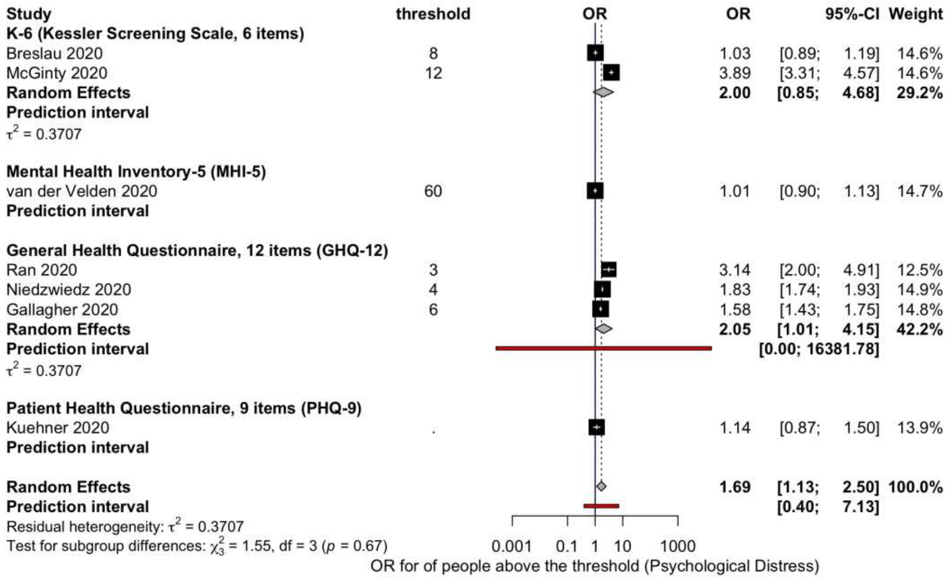

The forest plots indicate that, using short versions of scales (PHQ-2 and GAD-2 for depression and anxiety respectively) is associated with larger effect sizes that show important deterioration of the symptoms during the pandemic.

2.3 Sensitivity analyses including only adult populations and low risk of bias studies

The forest plot below shows the synthesis of studies on adult populations (excluding studies in older participants) by condition. Due to small number of studies excluded, there is no remarkable change in the summary estimates.

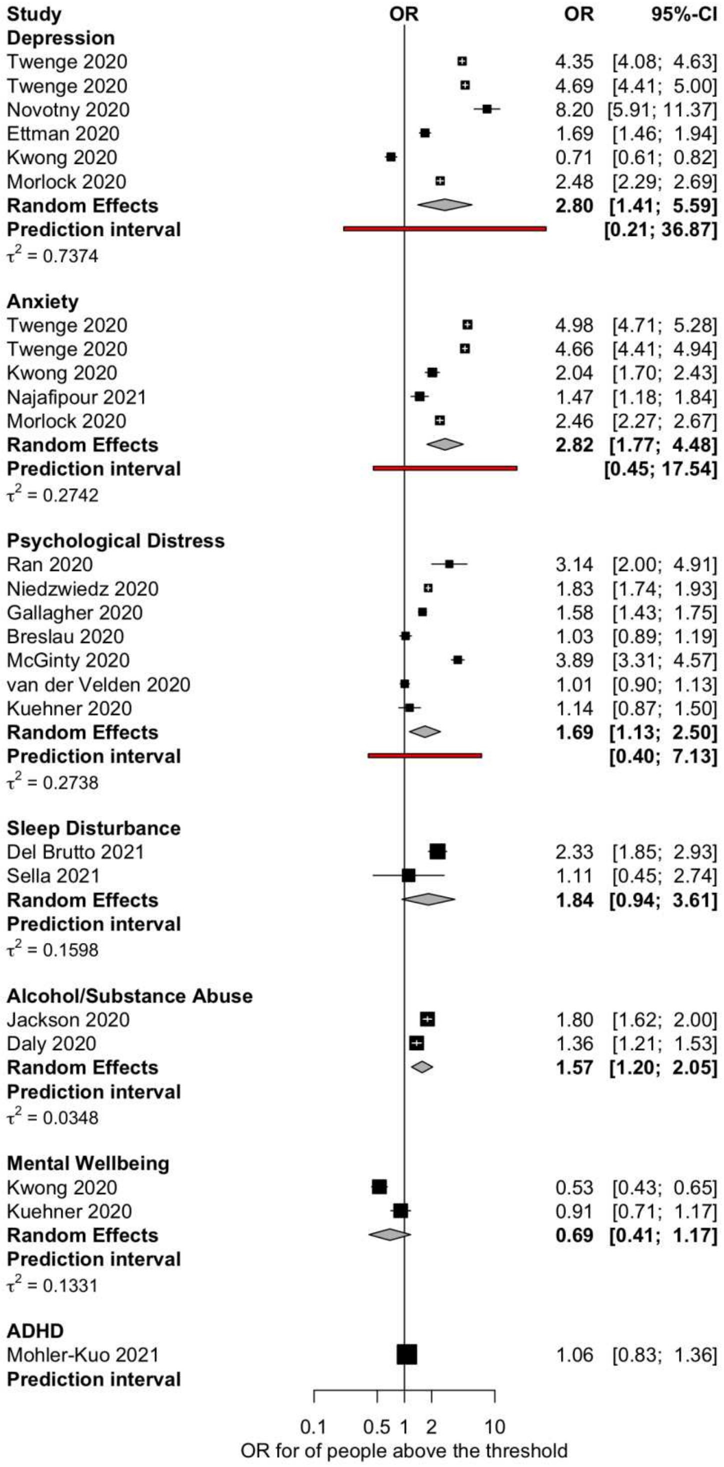
Study
OR
OR
95%-CI
Depression
Twenge 2020 4.35 [4.08; 4.63]
Twenge 2020 4.69 [4.41; 5.00]
Novotny 2020 8.20 [5.91; 11.37]
Ettman 2020 1.69 [1.46; 1.94]
Kwong 2020 0.71 [0.61; 0.82]
Morlock 2020 2.48 [2.29; 2.69]
Random Effects 2.80 [1.41; 5.59]
Prediction interval [0.21; 36.87]
τ2 = 0.7374
Anxiety
Twenge 2020 4.98 [4.71; 5.28]
Twenge 2020 4.66 [4.41; 4.94]
Kwong 2020 2.04 [1.70; 2.43]
Najafipour 2021 1.47 [1.18; 1.84]
Morlock 2020 2.46 [2.27; 2.67]
Random Effects 2.82 [1.77; 4.48]
Prediction interval [0.45; 17.54]
τ2 = 0.2742
Psychological Distress
Ran 2020 3.14 [2.00; 4.91]
Niedzwiedz 2020 1.83 [1.74; 1.93]
Gallagher 2020 1.58 [1.43; 1.75]
Breslau 2020 1.03 [0.89; 1.19]
McGinty 2020 3.89 [3.31; 4.57]
van der Velden 2020 1.01 [0.90; 1.13]
Kuehner 2020 1.14 [0.87; 1.50]
Random Effects 1.69 [1.13; 2.50]
Prediction interval [0.40; 7.13]
τ2 = 0.2738
Sleep Disturbance
Del Brutto 2021 2.33 [1.85; 2.93]
Sella 2021 1.11 [0.45; 2.74]
Random Effects 1.84 [0.94; 3.61]
Prediction interval
τ2 = 0.1598
Alcohol/Substance Abuse
Jackson 2020 1.80 [1.62; 2.00]
Daly 2020 1.36 [1.21; 1.53]
Random Effects 1.57 [1.20; 2.05]
Prediction interval
τ2 = 0.0348
Mental Wellbeing
Kwong 2020 0.53 [0.43; 0.65]
Kuehner 2020 0.91 [0.71; 1.17]
Random Effects 0.69 [0.41; 1.17]
Prediction interval
τ2 = 0.1331
ADHD
Mohler-Kuo 2021 1.06 [0.83; 1.36]
Prediction interval
0.1
0.5
1
2
10
OR for of people above the threshold

2.4 Funnel Plot

We included 10 studies that report data for depression.

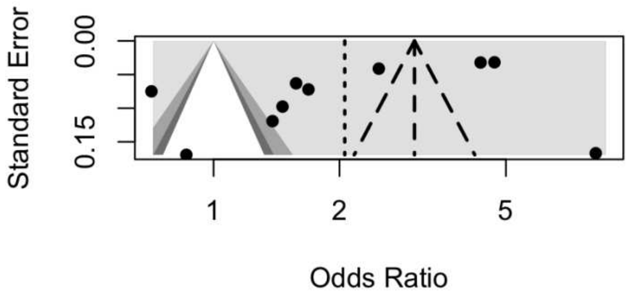

The funnel plot is not particularly asymmetric.

2.5 Meta-regression in studies for anxiety, depression, and psychological distress

We performed meta-regression analyses in the studies reporting results for depression, anxiety, and psychological distress by examining the role of age, percentage of female participants, country GDP as recorded in 2019 and the GINI index. The table below shows the unadjusted results for the summary ORs and the heterogeneity variance (assumed equal for the three conditions).

Meta-regression results for anxiety, depression, and psychological distress (pre-during analysis)

	No. of timepoints	OR	95% low CI	95% high CI	Heterogeneity variance
Anxiety	26	2.08	1.37	3.16	0.40
Depression	26	2.06	1.38	3.07	0.40
Psychological Distress	26	1.69	1.05	2.72	0.40
Mean age in years	17	1.00	0.99	1.01	0.34
Anxiety	17	1.51	0.89	2.58	0.34
Depression	17	1.57	0.98	2.50	0.34
Psychological Distress	17	1.56	0.89	2.73	0.34
Percentage of females	17	0.46	0.02	9.01	0.34
Anxiety	17	2.66	0.34	20.99	0.34
Depression	17	2.63	0.37	18.51	0.34
Psychological Distress	17	2.25	0.40	12.50	0.34
GDP per capita (in 1000$)	26	1.01	1.00	1.03	0.38
Anxiety	26	1.47	0.81	2.69	0.38

Meta-regression results for anxiety, depression, and psychological distress (pre-during analysis)

	No. of timepoints	OR	95% low CI	95% high CI	Heterogeneity variance
Depression	26	1.38	0.73	2.62	0.38
Psychological Distress	26	1.12	0.55	2.26	0.38
GINI index	26	1.01	0.97	1.06	0.41
Anxiety	26	1.75	0.81	3.79	0.41
Depression	26	1.76	0.87	3.58	0.41
Psychological Distress	26	1.47	0.73	2.98	0.41

Note:
ORs for condition refer to a study with average age of participants 30, GDP 12389 and GINI index 25

Accounting for age and sex in the model decreases the common heterogeneity parameter; however, the number of studies is small compared to the number of parameters estimated (five in total) to be able to draw any important conclusion about their role.

3 Results from dose-response meta-analysis

3.1 Dose-response depression as a function of days since the first recorded case

```
Call:  dosresmeta(formula = logOR ~ rcs(days_after_first, knots), id = reco
rd_id,
    type = type, cases = diagnosed, n = sample_size, data = dosedata,
    se = selogOR, proc = "1stage")

One-stage random-effects meta-analysis
Estimation method: REML
Covariance approximation: Greenland & Longnecker

Chi2 model: X2 = 158.3768 (df = 2), p-value = 0.0000

Fixed-effects coefficients
                                               Estimate  Std. Error
z  Pr(>|z|)  95%ci.lb  95%ci.ub
rcs(days_after_first, knots)days_after_first     0.0111      0.0033   3.335
7    0.0009    0.0046    0.0176  ***
rcs(days_after_first, knots)days_after_first'   -0.0064      0.0009  -7.158
6    0.0000   -0.0082   -0.0047  ***
---
Signif. codes:  0 '***' 0.001 '**' 0.01 '*' 0.05 '.' 0.1 ' ' 1

Between-study random-effects (co)variance components
                                               Std. Dev
Corr
rcs(days_after_first, knots)days_after_first     0.0120  rcs(days_after_fir
st, knots)days_after_first
rcs(days_after_first, knots)days_after_first'    0.0029
-1
```

```
14 studies, 16 values, 2 fixed and 3 random-effects parameters
  logLik       AIC       BIC
-19.9440   49.8879   53.0832
```

3.2 Dose-response depression as a function of stringency index

```
Call:  dosresmeta(formula = logOR ~ rcs(stringency, knots), id = record_id,
    type = type, cases = diagnosed, n = sample_size, data = dosedata,
    se = selogOR, proc = "1stage")

One-stage random-effects meta-analysis
Estimation method: REML
Covariance approximation: Greenland & Longnecker

Chi2 model: X2 = 6.1561 (df = 2), p-value = 0.0460

Fixed-effects coefficients
                                   Estimate  Std. Error        z  Pr(>|z|)
95%ci.lb  95%ci.ub
rcs(stringency, knots)stringency     0.0285      0.0146   1.9569    0.0504
-0.0000    0.0571  .
rcs(stringency, knots)stringency'   -0.0245      0.0141  -1.7374    0.0823
-0.0522    0.0031  .
---
Signif. codes:  0 '***' 0.001 '**' 0.01 '*' 0.05 '.' 0.1 ' ' 1

Between-study random-effects (co)variance components
                                   Std. Dev                              Co
rr
rcs(stringency, knots)stringency     0.0368  rcs(stringency, knots)stringen
cy
rcs(stringency, knots)stringency'    0.0316
-1

14 studies, 16 values, 2 fixed and 3 random-effects parameters
  logLik       AIC       BIC
-61.5271  133.0542  136.2495
```

3.3 Dose-response depression as a function of log-cases per 10.000 people

```
Call:  dosresmeta(formula = logOR ~ rcs(logconfirmed_cumulative100k,
    knots), id = record_id, type = type, cases = diagnosed, n = sample_size
,
    data = dosedata, se = selogOR, proc = "1stage")

One-stage random-effects meta-analysis
Estimation method: REML
Covariance approximation: Greenland & Longnecker

Chi2 model: X2 = 8.4413 (df = 2), p-value = 0.0147

Fixed-effects coefficients
                                                                    Estima
te  Std. Error        z  Pr(>|z|)  95%ci.lb
rcs(logconfirmed_cumulative100k, knots)logconfirmed_cumulative100k     0.32
75      0.1614   2.0295    0.0424    0.0112
rcs(logconfirmed_cumulative100k, knots)logconfirmed_cumulative100k'   -0.29
84      0.2133  -1.3993    0.1617   -0.7165
                                                                    95%ci.
ub
```

```
rcs(logconfirmed_cumulative100k, knots)logconfirmed_cumulative100k     0.64
37  *
rcs(logconfirmed_cumulative100k, knots)logconfirmed_cumulative100k'    0.11
96
---
Signif. codes:  0 ‘***’ 0.001 ‘**’ 0.01 ‘*’ 0.05 ‘.’ 0.1 ‘ ’ 1

Between-study random-effects (co)variance components
                                                                     Std. D
ev
rcs(logconfirmed_cumulative100k, knots)logconfirmed_cumulative100k     0.44
52
rcs(logconfirmed_cumulative100k, knots)logconfirmed_cumulative100k'    0.54
16

Corr
rcs(logconfirmed_cumulative100k, knots)logconfirmed_cumulative100k   rcs(lo
gconfirmed_cumulative100k, knots)logconfirmed_cumulative100k
rcs(logconfirmed_cumulative100k, knots)logconfirmed_cumulative100k'
-0.9723

14 studies, 16 values, 2 fixed and 3 random-effects parameters
 logLik      AIC      BIC
-9.7018  29.4036  32.5989
```

3.4 Dose-response depression as a function of log-deaths per 10.000 people

```
Call:  dosresmeta(formula = logOR ~ rcs(logdeaths_cumulative100k, knots),
    id = record_id, type = type, cases = diagnosed, n = sample_size,
    data = dosedata, se = selogOR, proc = "1stage")

One-stage random-effects meta-analysis
Estimation method: REML
Covariance approximation: Greenland & Longnecker

Chi2 model: X2 = 17.3779 (df = 2), p-value = 0.0002

Fixed-effects coefficients
                                                               Estimate  St
d. Error        z  Pr(>|z|)  95%ci.lb
rcs(logdeaths_cumulative100k, knots)logdeaths_cumulative100k     1.1796
0.3107   3.7971    0.0001    0.5707
rcs(logdeaths_cumulative100k, knots)logdeaths_cumulative100k'   -6.1933
1.9125  -3.2384    0.0012   -9.9417
                                                               95%ci.ub
rcs(logdeaths_cumulative100k, knots)logdeaths_cumulative100k     1.7885  **
*
rcs(logdeaths_cumulative100k, knots)logdeaths_cumulative100k'   -2.4450   *
*
---
Signif. codes:  0 ‘***’ 0.001 ‘**’ 0.01 ‘*’ 0.05 ‘.’ 0.1 ‘ ’ 1

Between-study random-effects (co)variance components
                                                               Std. Dev
rcs(logdeaths_cumulative100k, knots)logdeaths_cumulative100k     0.7839
rcs(logdeaths_cumulative100k, knots)logdeaths_cumulative100k'    4.7223

Corr
rcs(logdeaths_cumulative100k, knots)logdeaths_cumulative100k   rcs(logdeath
s_cumulative100k, knots)logdeaths_cumulative100k
```

```
rcs(logdeaths_cumulative100k, knots)logdeaths_cumulative100k'
-0.9698

14 studies, 16 values, 2 fixed and 3 random-effects parameters
  logLik       AIC       BIC
-12.4192   34.8383   38.0336
```

3.5 Dose-response anxiety as a function of days since the first recorded case

```
Call:  dosresmeta(formula = logOR ~ rcs(days_after_first, knots), id = reco
rd_id,
    type = type, cases = diagnosed, n = sample_size, data = dosedata,
    se = selogOR, proc = "1stage")

One-stage random-effects meta-analysis
Estimation method: REML
Covariance approximation: Greenland & Longnecker

Chi2 model: X2 = 912.8713 (df = 2), p-value = 0.0000

Fixed-effects coefficients
                                               Estimate  Std. Error
z  Pr(>|z|)  95%ci.lb  95%ci.ub
rcs(days_after_first, knots)days_after_first     0.0156      0.0017    9.14
71    0.0000    0.0123    0.0190  ***
rcs(days_after_first, knots)days_after_first'   -0.0122      0.0006  -21.72
32    0.0000   -0.0133   -0.0111  ***
---
Signif. codes:  0 '***' 0.001 '**' 0.01 '*' 0.05 '.' 0.1 ' ' 1

Between-study random-effects (co)variance components
                                               Std. Dev
Corr
rcs(days_after_first, knots)days_after_first     0.0056  rcs(days_after_fir
st, knots)days_after_first
rcs(days_after_first, knots)days_after_first'    0.0014
-1

13 studies, 15 values, 2 fixed and 3 random-effects parameters
  logLik       AIC       BIC
-12.0314   34.0627   36.8875
```

3.6 Dose-response anxiety as a function of stringency index

```
Call:  dosresmeta(formula = logOR ~ rcs(stringency, knots), id = record_id,
    type = type, cases = diagnosed, n = sample_size, data = dosedata,
    se = selogOR, proc = "1stage")

One-stage random-effects meta-analysis
Estimation method: REML
Covariance approximation: Greenland & Longnecker

Chi2 model: X2 = 15.6984 (df = 2), p-value = 0.0004

Fixed-effects coefficients
                                   Estimate  Std. Error       z  Pr(>|z|)
95%ci.lb  95%ci.ub
rcs(stringency, knots)stringency     0.0086      0.0059  1.4557    0.1455
-0.0030    0.0201
```

```
rcs(stringency, knots)stringency'    0.0002      0.0058  0.0346    0.9724
-0.0112    0.0116
---
Signif. codes:  0 ‘***’ 0.001 ‘**’ 0.01 ‘*’ 0.05 ‘.’ 0.1 ‘ ’ 1

Between-study random-effects (co)variance components
                                   Std. Dev                             Co
rr
rcs(stringency, knots)stringency     0.0027  rcs(stringency, knots)stringen
cy
rcs(stringency, knots)stringency'    0.0045
1

13 studies, 15 values, 2 fixed and 3 random-effects parameters
  logLik       AIC       BIC
-45.8208  101.6415  104.4663
```

3.7 Dose-response anxiety as a function of log-cases per 10.000 people

```
Call:  dosresmeta(formula = logOR ~ rcs(logconfirmed_cumulative100k,
    knots), id = record_id, type = type, cases = diagnosed, n = sample_size
,
    data = dosedata, se = selogOR, proc = "1stage")

One-stage random-effects meta-analysis
Estimation method: REML
Covariance approximation: Greenland & Longnecker

Chi2 model: X2 = 140.3727 (df = 2), p-value = 0.0000

Fixed-effects coefficients
                                                                    Estima
te  Std. Error        z  Pr(>|z|)  95%ci.lb
rcs(logconfirmed_cumulative100k, knots)logconfirmed_cumulative100k     0.43
01      0.0789   5.4546    0.0000    0.2756
rcs(logconfirmed_cumulative100k, knots)logconfirmed_cumulative100k'   -0.63
65      0.0794  -8.0184    0.0000   -0.7921
                                                                    95%ci.
ub
rcs(logconfirmed_cumulative100k, knots)logconfirmed_cumulative100k     0.58
47  ***
rcs(logconfirmed_cumulative100k, knots)logconfirmed_cumulative100k'   -0.48
09  ***
---
Signif. codes:  0 ‘***’ 0.001 ‘**’ 0.01 ‘*’ 0.05 ‘.’ 0.1 ‘ ’ 1

Between-study random-effects (co)variance components
                                                                    Std. D
ev
rcs(logconfirmed_cumulative100k, knots)logconfirmed_cumulative100k     0.22
44
rcs(logconfirmed_cumulative100k, knots)logconfirmed_cumulative100k'    0.20
16

Corr
rcs(logconfirmed_cumulative100k, knots)logconfirmed_cumulative100k   rcs(lo
gconfirmed_cumulative100k, knots)logconfirmed_cumulative100k
rcs(logconfirmed_cumulative100k, knots)logconfirmed_cumulative100k'
-1

13 studies, 15 values, 2 fixed and 3 random-effects parameters
```


Salanti G, *et al. BMJ Ment Health* 2024; 27:e301018. doi: 10.1136/bmjment-2024-301018

```
  logLik       AIC       BIC
-21.2398   52.4796   55.3044
```

3.8 Dose-response anxiety as a function of log-deaths per 10.000 people

```
Call:  dosresmeta(formula = logOR ~ rcs(logdeaths_cumulative100k, knots), 
    id = record_id, type = type, cases = diagnosed, n = sample_size, 
    data = dosedata, se = selogOR, proc = "1stage")

One-stage random-effects meta-analysis
Estimation method: REML
Covariance approximation: Greenland & Longnecker

Chi2 model: X2 = 28.7511 (df = 2), p-value = 0.0000

Fixed-effects coefficients
                                                                    Estimate  St
d. Error        z  Pr(>|z|)  95%ci.lb
rcs(logdeaths_cumulative100k, knots)logdeaths_cumulative100k          1.4907    
0.3298   4.5199    0.0000    0.8443
rcs(logdeaths_cumulative100k, knots)logdeaths_cumulative100k'  -12.1081    
3.0492  -3.9709    0.0001  -18.0844
                                                                    95%ci.ub    
rcs(logdeaths_cumulative100k, knots)logdeaths_cumulative100k     2.1372  **
*
rcs(logdeaths_cumulative100k, knots)logdeaths_cumulative100k'   -6.1318  **
*
---
Signif. codes:  0 '***' 0.001 '**' 0.01 '*' 0.05 '.' 0.1 ' ' 1

Between-study random-effects (co)variance components
                                                                    Std. Dev
rcs(logdeaths_cumulative100k, knots)logdeaths_cumulative100k     0.7375
rcs(logdeaths_cumulative100k, knots)logdeaths_cumulative100k'    6.7796

Corr
rcs(logdeaths_cumulative100k, knots)logdeaths_cumulative100k   rcs(logdeath
s_cumulative100k, knots)logdeaths_cumulative100k
rcs(logdeaths_cumulative100k, knots)logdeaths_cumulative100k'
-0.9865

13 studies, 15 values, 2 fixed and 3 random-effects parameters
  logLik       AIC       BIC
-18.1105   46.2210   49.0458
```

3.9 Dose-response psychological distress as a function of days since the first recorded case

```
Call:  dosresmeta(formula = logOR ~ rcs(days_after_first, knots), id = reco
rd_id, 
    type = type, cases = diagnosed, n = sample_size, data = dosedata, 
    se = selogOR, proc = "1stage")

One-stage random-effects meta-analysis
Estimation method: REML
Covariance approximation: Greenland & Longnecker

Chi2 model: X2 = 7.7960 (df = 2), p-value = 0.0203

Fixed-effects coefficients
```

```
                                               Estimate  Std. Error
z  Pr(>|z|)  95%ci.lb  95%ci.ub
rcs(days_after_first, knots)days_after_first     0.0088      0.0038   2.318
6    0.0204    0.0014    0.0163   *
rcs(days_after_first, knots)days_after_first'   -0.0080      0.0030  -2.706
0    0.0068   -0.0138   -0.0022  **
---
Signif. codes:  0 '***' 0.001 '**' 0.01 '*' 0.05 '.' 0.1 ' ' 1

Between-study random-effects (co)variance components
                                               Std. Dev
Corr
rcs(days_after_first, knots)days_after_first     0.0119  rcs(days_after_fir
st, knots)days_after_first
rcs(days_after_first, knots)days_after_first'    0.0091
-0.983

12 studies, 15 values, 2 fixed and 3 random-effects parameters
  logLik       AIC       BIC
-16.9954   43.9907   46.8155
```

3.10 Dose-response psychological distress as a function of stringency index

```
Call:  dosresmeta(formula = logOR ~ rcs(stringency, knots), id = record_id,
    type = type, cases = diagnosed, n = sample_size, data = dosedata,
    se = selogOR, proc = "1stage")

One-stage random-effects meta-analysis
Estimation method: REML
Covariance approximation: Greenland & Longnecker

Chi2 model: X2 = 15.0616 (df = 2), p-value = 0.0005

Fixed-effects coefficients
                                   Estimate  Std. Error        z  Pr(>|z|)
95%ci.lb  95%ci.ub
rcs(stringency, knots)stringency     0.0163      0.0105   1.5531    0.1204
-0.0043    0.0369
rcs(stringency, knots)stringency'   -0.0067      0.0094  -0.7119    0.4765
-0.0251    0.0117
---
Signif. codes:  0 '***' 0.001 '**' 0.01 '*' 0.05 '.' 0.1 ' ' 1

Between-study random-effects (co)variance components
                                   Std. Dev                              Co
rr
rcs(stringency, knots)stringency     0.0293  rcs(stringency, knots)stringen
cy
rcs(stringency, knots)stringency'    0.0222
-1

12 studies, 15 values, 2 fixed and 3 random-effects parameters
  logLik       AIC       BIC
-28.8414   67.6827   70.5075
```

3.11 Dose-response psychological distress as a function of log-cases per 10.000 people

```
Call:  dosresmeta(formula = logOR ~ rcs(logconfirmed_cumulative100k,
    knots), id = record_id, type = type, cases = diagnosed, n = sample_size
,
```

```
    data = dosedata, se = selogOR, proc = "1stage")

One-stage random-effects meta-analysis
Estimation method: REML
Covariance approximation: Greenland & Longnecker

Chi2 model: X2 = 8.9454 (df = 2), p-value = 0.0114

Fixed-effects coefficients
                                                                           Estima
te  Std. Error        z  Pr(>|z|)  95%ci.lb
rcs(logconfirmed_cumulative100k, knots)logconfirmed_cumulative100k     0.22
86      0.0765   2.9896    0.0028    0.0787
rcs(logconfirmed_cumulative100k, knots)logconfirmed_cumulative100k'   -0.14
86      0.0575  -2.5848    0.0097   -0.2612
                                                                           95%ci.
ub
rcs(logconfirmed_cumulative100k, knots)logconfirmed_cumulative100k     0.37
85  **
rcs(logconfirmed_cumulative100k, knots)logconfirmed_cumulative100k'   -0.03
59  **
---
Signif. codes:  0 '***' 0.001 '**' 0.01 '*' 0.05 '.' 0.1 ' ' 1

Between-study random-effects (co)variance components
                                                                           Std. D
ev
rcs(logconfirmed_cumulative100k, knots)logconfirmed_cumulative100k     0.25
53
rcs(logconfirmed_cumulative100k, knots)logconfirmed_cumulative100k'    0.17
68

Corr
rcs(logconfirmed_cumulative100k, knots)logconfirmed_cumulative100k   rcs(lo
gconfirmed_cumulative100k, knots)logconfirmed_cumulative100k
rcs(logconfirmed_cumulative100k, knots)logconfirmed_cumulative100k'
-1

12 studies, 15 values, 2 fixed and 3 random-effects parameters
  logLik       AIC       BIC
-65.9007  141.8014  144.6261
```

3.12 Dose-response psychological distress as a function of log-deaths per 10.000 people

```
Call:  dosresmeta(formula = logOR ~ rcs(logdeaths_cumulative100k, knots),
    id = record_id, type = type, cases = diagnosed, n = sample_size,
    data = dosedata, se = selogOR, proc = "1stage")

One-stage random-effects meta-analysis
Estimation method: REML
Covariance approximation: Greenland & Longnecker

Chi2 model: X2 = 7.4615 (df = 2), p-value = 0.0240

Fixed-effects coefficients
                                                                     Estimate  St
d. Error        z  Pr(>|z|)  95%ci.lb
rcs(logdeaths_cumulative100k, knots)logdeaths_cumulative100k     0.4323
0.1592   2.7150    0.0066    0.1202
rcs(logdeaths_cumulative100k, knots)logdeaths_cumulative100k'   -0.3733
0.1414  -2.6403    0.0083   -0.6504
```

```
                                                                95%ci.ub
rcs(logdeaths_cumulative100k, knots)logdeaths_cumulative100k      0.7445  **
rcs(logdeaths_cumulative100k, knots)logdeaths_cumulative100k'    -0.0962  **
---
Signif. codes:  0 '***' 0.001 '**' 0.01 '*' 0.05 '.' 0.1 ' ' 1

Between-study random-effects (co)variance components
                                                                Std. Dev
rcs(logdeaths_cumulative100k, knots)logdeaths_cumulative100k      0.4517
rcs(logdeaths_cumulative100k, knots)logdeaths_cumulative100k'     0.4215

Corr
rcs(logdeaths_cumulative100k, knots)logdeaths_cumulative100k   rcs(logdeath
s_cumulative100k, knots)logdeaths_cumulative100k
rcs(logdeaths_cumulative100k, knots)logdeaths_cumulative100k'
-1

12 studies, 15 values, 2 fixed and 3 random-effects parameters
  logLik       AIC       BIC
-81.0987  172.1973  175.0221
```

4 Sensitivity analysis for dose-response meta-analysis

4.1 Excluding a study with very long follow-up

In dose-response associated for depression and anxiety there was a study by Herrera 2021 with follow-up 252 days. The following graphs show the updated dose-response associations when this study is excluded from the analysis.

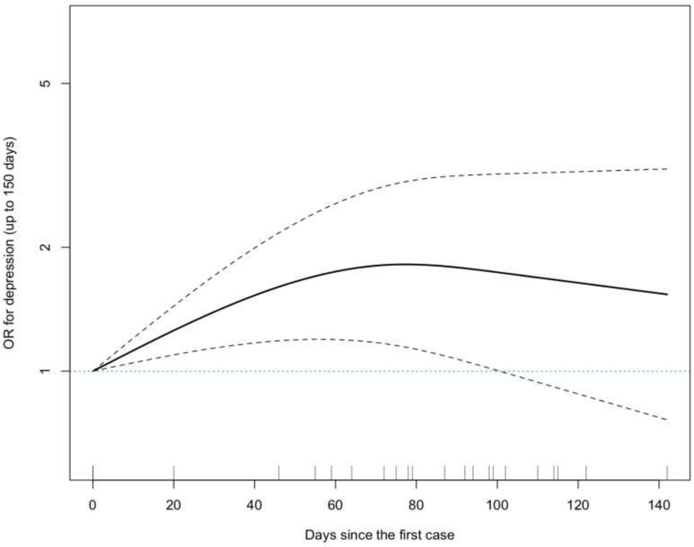

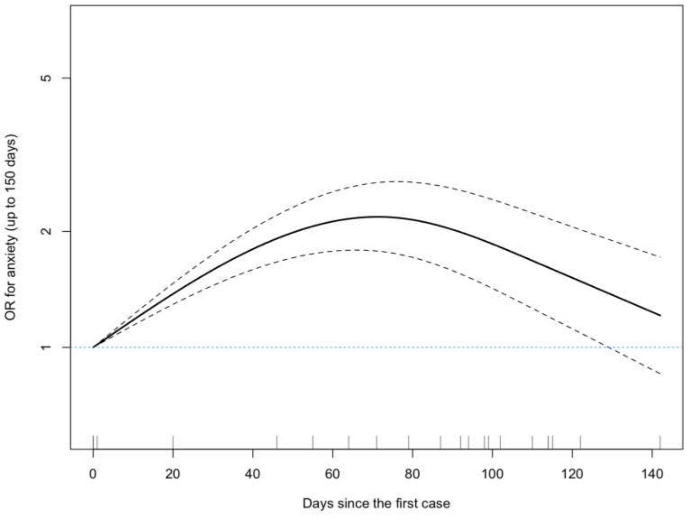

4.2 Sensitivity to knot locations

We chose two different knot location: closer to the median and more far apart from the median. Due to the small number of studies, we could not explore the role of the number of knots.

4.3 Knots at 30%, 50% and 70% quintiles

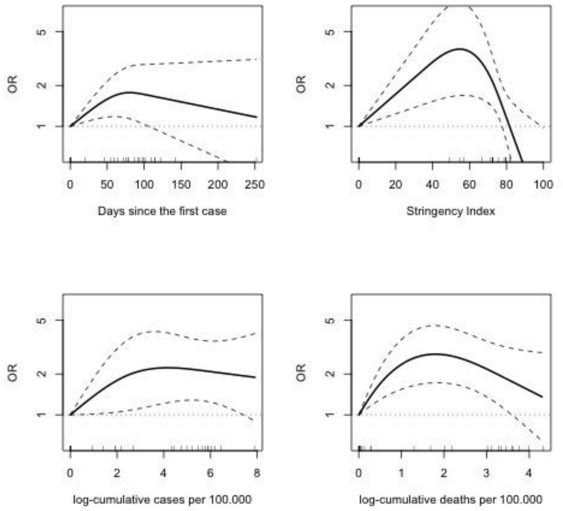

Appendix Figure *Dose-response meta-analysis plots of the odds ratios (OR) for depression as function of the days since the days of the first case in the study country, the stringency index, the cumulative number of cases and the cumulative number of deaths. Confidence intervals are shown as dashed lines. Larger ORs mean that the number of people above the threshold increases over time.*

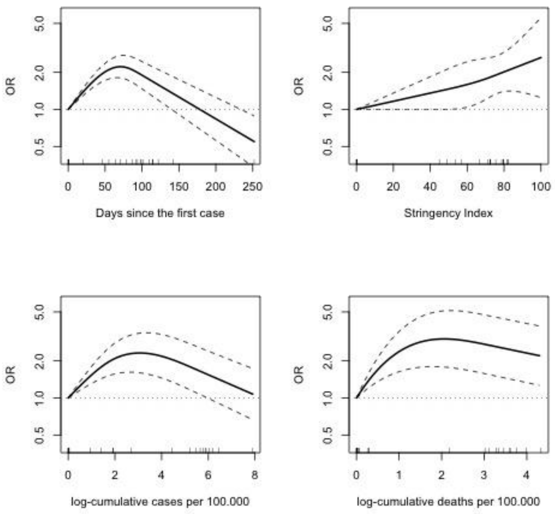

Appendix Figure *Dose-response meta-analysis plots of the odds ratios (OR) for anxiety as a function of the days since the days of the first case in the study country, the stringency index, the cumulative number of cases and the cumulative number of deaths. Confidence intervals are shown as dashed lines. Larger ORs mean that the number of people above the threshold increases over time.*

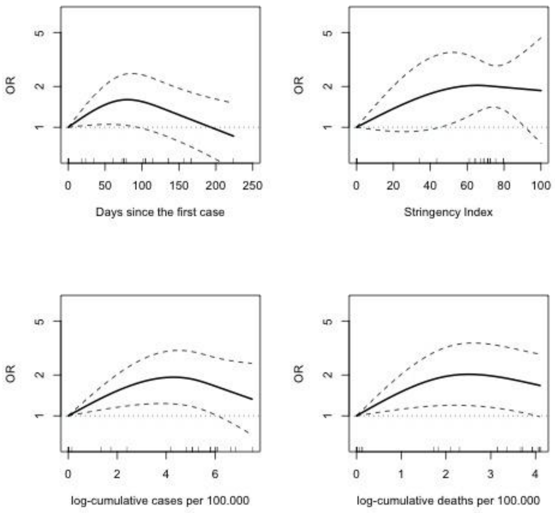

Appendix Figure *Dose-response meta-analysis plots of the odds ratios (OR) for psychological distress as a function of the days since the days of the first case in the study country, the stringency index, the cumulative number of cases and the cumulative number of deaths. Confidence intervals are shown as dashed lines. Larger ORs mean that the number of people above the threshold increases over time.*

4.4 Knots at 10%, 50% and 90% quintiles

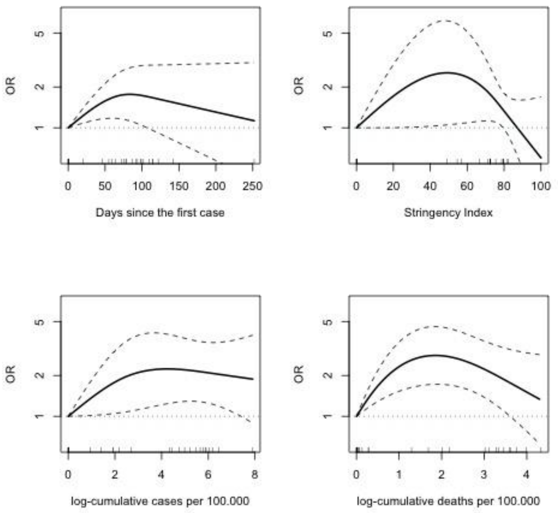

Appendix Figure *Dose-response meta-analysis plots of the odds ratios (OR) for depression as function of the days since the days of the first case in the study country, the stringency index, the cumulative number of cases and the cumulative number of deaths. Confidence intervals are shown as dashed lines. Larger ORs mean that the number of people above the threshold increases over time.*

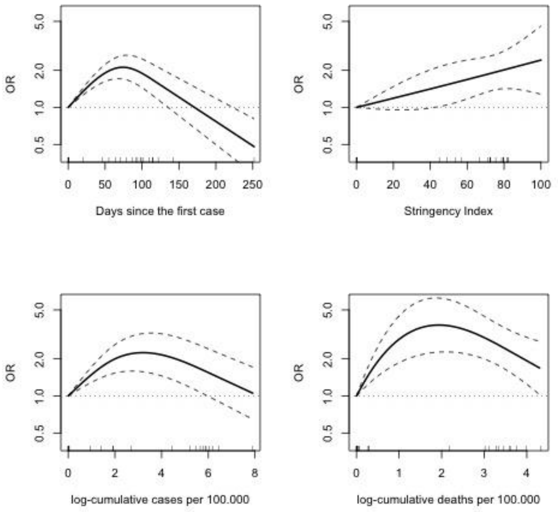

Appendix Figure *Dose-response meta-analysis plots of the odds ratios (OR) for anxiety as a function of the days since the days of the first case in the study country, the stringency index, the cumulative number of cases and the cumulative number of deaths. Confidence intervals are shown as dashed lines. Larger ORs mean that the number of people above the threshold increases over time.*

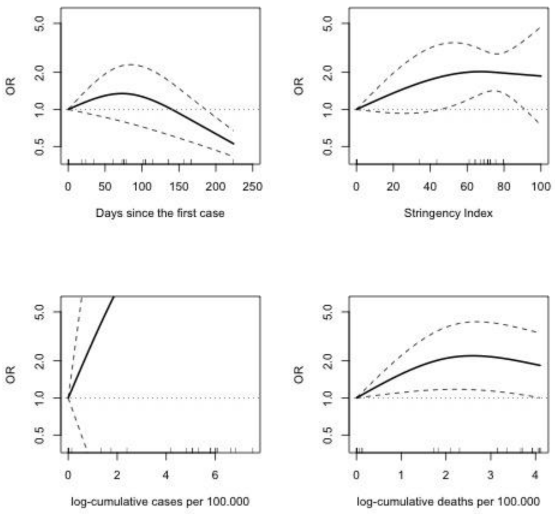

Appendix Figure *Dose-response meta-analysis plots of the odds ratios (OR) for psychological distress as a function of the days since the days of the first case in the study country, the stringency index, the cumulative number of cases and the cumulative number of deaths. Confidence intervals are shown as dashed lines. Larger ORs mean that the number of people above the threshold increases over time.*

5 Included studies and their characteristics.

condition	doi	author & year	country	symptomes scale	threshold	population	study design	study included in previous article*
Anxiety	10.1002/da.23077	Twenge 2020	United States	Generalized Anxiety Disorder Scale, 2 items (GAD-2)	3	Adults	Longitudinal	no
Depression	10.1002/da.23077	Twenge 2020	United States	Patient Health Questionnaire, 2 items (PHQ-2)	3	Adults	Longitudinal	no
Depression	10.1177/0706743720943812	Schmitz 2020	Canada	Patient Health Questionnaire, 9 items pre/8 during	10	Adults	Longitudinal	no
Sleep Disturbance	10.1111/jsr.13142	Liu 2020	China	Children's Sleep Habit Questionnaire (CSHQ)	42	Children	Longitudinal	yes
Psychological Distress	10.1001/jama.2020.9740	McGinty 2020	United States	K-6 (Kessler Screening Scale, 6 items)	12	Adults	Longitudinal	no
Depression	10.1001/jamanetworkopen.2020.19686	Ettman 2020	United States	Patient Health Questionnaire, 9 items (PHQ-9)	5	Adults	Longitudinal	no
Psychological Distress	10.1016/j.jad.2020.08.026	van der Velden 2020	Netherlands	Mental Health Inventory-5 (MHI-5)	60	Adults	Cross-sectional	yes
Anxiety	10.1001/jamanetworkopen.2020.21482	Zhang 2020	China	HBQ anxiety subscale	4.17	Children	Cross-sectional	no
Depression	10.1001/jamanetworkopen.2020.21482	Zhang 2020	China	Mood and Feelings Questionnaire (MFQ)	27	Children	Cross-sectional	no
Anxiety	10.1038/s41380-020-00881-6	Wang 2020	China	Generalized Anxiety Disorder Scale, 7 items (GAD-7)	5	Adults	Longitudinal	yes
Depression	10.1038/s41380-020-00881-6	Wang 2020	China	Patient Health Questionnaire, 9 items (PHQ-9)	5	Adults	Longitudinal	yes
Depression	10.1016/j.jad.2020.09.065	Daly 2020	United States	Patient Health Questionnaire, 2 items (PHQ-2)	3	Adults	Longitudinal	no
Psychological Distress	10.2188/jea.JE20200271	Kikuchi 2020	Japan	Kessler Psychological Distress Scale, 6 items (K-6)	13	Adults	Cross-sectional	yes
Psychological Distress	10.1136/jech-2020-215060	Niedzwiedz 2020	United Kingdom	General Health Questionnaire, 12 items (GHQ-12)	4	Adults	Cross-sectional	no
Psychological Distress	10.1192/bjo.2020.99	Gallagher 2020	United Kingdom	General Health Questionnaire, 12 items (GHQ-12)	6	Adults	Cross-sectional	no

Anxiety	10.1192/bjp.2020.212	O'Connor 2020	United Kingdom	Generalized Anxiety Disorder Scale, 7 items (GAD-7)	10	Adults	Cross-sectional	yes
Depression	10.1192/bjp.2020.212	O'Connor 2020	United Kingdom	Patient Health Questionnaire, 9 items (PHQ-9)	10	Adults	Cross-sectional	yes
Alcohol/Substance Abuse	10.1111/add.15295	Jackson 2020	United Kingdom	Alcohol Use Disorder Identification Test Consumption (AUDIT-C)	5	Adults	Longitudinal	no
Anxiety	10.3760/cma.j.cn112137-20200720-02167	Wang 2020	China	Generalized Anxiety Disorder Scale, 2 items (GAD-2)	2	Elderly	Cross-sectional	no
Anxiety	10.1002/da.23109	Kujawa 2020	United States	Generalized Anxiety Disorder Scale, 7 items (GAD-7)	10	Adults	Cross-sectional	yes
Depression	10.1002/da.23109	Kujawa 2020	United States	Patient Health Questionnaire, 9 items (PHQ-9)	10	Adults	Cross-sectional	yes
Psychological Distress	10.1017/S0033291720004432	Daly 2020	United Kingdom	General Health Questionnaire, 12 items (GHQ-12)	3	Adults	Cross-sectional	no
Psychological Distress	10.1001/jama.2020.21231	McGinty 2020	United States	K-6 (Kessler Screening Scale, 6 items)	13	Adults	Cross-sectional	no
Psychological Distress	10.1017/S0033291720005048	Chandola 2020	United Kingdom	General Health Questionnaire, 12 items (GHQ-12)	3	Adults	Cross-sectional	no
Psychological Distress	10.1017/S0033291720004717	Ran 2020	China	General Health Questionnaire, 12 items (GHQ-12)	3	Adults	Longitudinal	no
Anxiety	10.1016/j.jval.2020.08.1103	Morlock 2020	United States	Generalized Anxiety Disorder Scale, 7 items (GAD-7)	NA	Adults	Longitudinal	no
Depression	10.1016/j.jval.2020.08.1103	Morlock 2020	United States	Patient Health Questionnaire, 9 items (PHQ-9)	NA	Adults	Longitudinal	no
Anxiety	10.1017/S1041610220004135	Creese 2020	United Kingdom	Generalized Anxiety Disorder Scale, 7 items (GAD-7)	5	Elderly	Cross-sectional	no
Depression	10.1017/S1041610220004135	Creese 2020	United Kingdom	Patient Health Questionnaire, 9 items (PHQ-9)	5	Elderly	Cross-sectional	no
Alcohol/Substance Abuse	10.1016/j.psychres.2020.113676	Killgore 2020	United States	Alcohol Use Disorders Identification Test (AUDIT)	8	Adults	Longitudinal	no
Depression	10.3389/fpsyt.2020.603014	Novotny 2020	Czechia	Patient Health Questionnaire, 2 items (PHQ-2)	3	Adults	Cross-sectional	yes
Psychological Distress	10.1192/bjo.2021.5	Kikuchi 2021	Japan	K-6 (Kessler Screening Scale, 6 items)	13	Adults	Cross-sectional	no
Psychological Distress	10.1001/jamanetworkopen.2020.37665	Czeisler 2021	United States	Patient Health Questionnaire, 4 items (PHQ-4)	3	Adults	Longitudinal	no
Mental Wellbeing	10.1055/a-1222-9067	Kuehner 2020	Germany	WHO-5 Wellbeing Index (WHO-5)	50	Adults	Longitudinal	yes
Psychological Distress	10.1055/a-1222-9067	Kuehner 2020	Germany	Patient Health Questionnaire, 9 items (PHQ-9)	NA	Adults	Longitudinal	yes

Sleep Disturbance	10.1093/sleep/zsab041	Del Brutto 2021	Ecuador	Pittsburgh Sleep Quality Index (PSQI)	5	Adults	Cross-sectional	no
Anxiety	10.1186/s12877-021-02110-3	Herrera 2021	Chile	Geriatric Anxiety Inventory, short form (GAI-SF)	3	Elderly	Cross-sectional	yes
Depression	10.1186/s12877-021-02110-3	Herrera 2021	Chile	Patient Health Questionnaire, 9 items (PHQ-9)	7	Elderly	Cross-sectional	yes
Psychological Distress	10.3390/ijerph18052342	Hakansson 2021	Sweden	K-6 (Kessler Screening Scale, 6 items)	5	Adults	Longitudinal	no
Sleep Disturbance	10.1016/j.sleep.2021.02.017	Sella 2021	Italy	Pittsburgh Sleep Quality Index (PSQI)	5	Adults	Cross-sectional	yes
Sleep Disturbance	10.1093/ageing/afab061	Wang 2021	China	Pittsburgh Sleep Quality Index (PSQI)	7	Elderly	Cross-sectional	yes
Anxiety	10.1038/s41398-021-01279-w	Hou 2021	Hong Kong	Generalized Anxiety Disorder Scale, 7 items (GAD-7)	10	Adolescents,Adults	Longitudinal	no
Depression	10.1038/s41398-021-01279-w	Hou 2021	Hong Kong	Patient Health Questionnaire, 9 items (PHQ-9)	10	Adolescents,Adults	Longitudinal	no
ADHD	10.3390/ijerph18094668	Mohler-Kuo 2021	Switzerland	Adult ADHD Self-Report Scale Screener (ASRS-v1.1)	14	Adults	Longitudinal	no
Alcohol/Substance Abuse	10.3390/ijerph18157849	Roges 2021	Spain	Alcohol Use Disorder Identification Test-Consumption (AUDIT-C)	3	Adolescents	Cross-sectional	no
Anxiety	10.22062/jkmu.2021.91661	Najafipour 2021	Iran	Beck Anxiety Inventory (BAI)	15	Adults	Cross-sectional	no
Mental Wellbeing	10.1007/s00787-021-01889-1	Ravens-Sieberer 2021	Germany	Strengths and Difficulties Questionnaire (SDQ)	13	Children,Adolescents	Cross-sectional	no
Sleep Disturbance	https://dx.doi.org/10.1111/jsr.13235	Cheng 2021	United Kingdom	PROMIS Sleep Disturbance short form T-score	55	Adults	Cross-sectional	no
Anxiety	https://dx.doi.org/10.1007/s11136-021-02861-x	Luijten 2021	Netherlands	PROMIS Anxiety T-score	NA	Children,Adolescents	Longitudinal	no
Depression	https://dx.doi.org/10.1007/s11136-021-02861-x	Luijten 2021	Netherlands	PROMIS Depressive Symptoms T-score	NA	Children,Adolescents	Longitudinal	no
Sleep Disturbance	https://dx.doi.org/10.1007/s11136-021-02861-x	Luijten 2021	Netherlands	PROMIS Sleep Related Impairment T-score	NA	Children,Adolescents	Longitudinal	no
Sleep Disturbance	https://dx.doi.org/10.1111/jsr.13235	Cheng 2021	United States	PROMIS Sleep Disturbance T-score	55	Adults	Cross-sectional	no

*included in a study of continuous outcomes in https://doi.org/10.7326/M22-150

6 Changes to the protocol

The protocol of this review is registered with PROSPERO CRD42020180049 (Available from: https://www.crd.york.ac.uk/prospero/display_record.php?ID=CRD42020180049).

In the protocol, we set two aims. We describe that we will “provide summaries of *a)* the prevalence of mental health issues, including alcohol/substance abuse and violent/aggressive behaviour and *b)* the change in mental health symptoms in the general population in relation to the COVID-19 epidemic/pandemic outbreak “

The current review presents results only for the first aim: the second aim (continuous mental health outcomes) was addressed in our article Salanti G, Peter N, Tonia T et al. MHCOVID Crowd Investigators. The Impact of the COVID-19 Pandemic and Associated Control Measures on the Mental Health of the General Population : A Systematic Review and Dose-Response Meta-analysis. Ann Intern Med. 2022 Nov;175(11):1560-1571.

Additionally, we decided not to include studies reporting on a single timepoint described in the “Types of studies to be included” in the protocol, as their risk of bias to inform about changes (after marching their results to pre-pandemic prevalence studies) with was deemed very high.

7 Examples of excluded studies

Ostertun Geirdal (2021), The significance of demographic variables on psychosocial health from the early stage and nine months after the covid-19 pandemic outbreak. A cross-national study. 10.3390/ijerph18084345 Excluded because of recruitment via social media

Al-Musharaf (2021), Lifestyle changes associated with COVID-19 quarantine among young Saudi women: A prospective study. 10.1371/journal.pone.0250625 Excluded because of the study population (university students)

Mary-Krause (2021), Impact of COVID-19-like symptoms on occurrence of anxiety/depression during lockdown among the French general population. 10.1371/journal.pone.0255158 Excluded because the instrument used was not validated.
